# Cell-penetrating interactomic inhibition of nuclear factor-kappa B in a mouse model of postoperative cognitive dysfunction

**DOI:** 10.1038/s41598-017-14027-2

**Published:** 2017-10-18

**Authors:** So Yeong Cheon, Jeong Min Kim, Eun Hee Kam, Chun-Chang Ho, Eun Jung Kim, Seungsoo Chung, Ji-Hyun Jeong, Diane Da-Hyun Lee, Sang-Won Lee, Bon-Nyeo Koo

**Affiliations:** 1 Department of Anesthesiology and Pain Medicine, Yonsei University College of Medicine, Seoul, Republic of Korea; 2 Anesthesia and Pain Research Institute, Yonsei University College of Medicine, Seoul, Republic of Korea; 3 Department of Biotechnology, College of Life Science and Biotechnology, Yonsei University, Seoul, Republic of Korea; 4 Department of Physiology, Brain Korea 21 Plus Project for Medical Science, Yonsei University College of Medicine, Seoul, Republic of Korea; 5 Division of Rheumatology, Department of Internal Medicine, Yonsei University College of Medicine, Seoul, Republic of Korea; 6 Seoul International School, Seongnam, Republic of Korea

## Abstract

Some patients experience impaired cognitive functioning after surgery, a phenomenon referred to as postoperative cognitive dysfunction (POCD). Signs of POCD are closely associated with the development of systemic or hippocampal inflammation. However, the precise pathophysiological mechanisms of prevention/treatment options for POCD still remain unclear. After injury, the transcriptional factor nuclear factor-kappa B (NF-κB) is thought to regulate or stimulate inflammation amplification. Therefore, we designed a cell-penetrating fusion protein called nt-p65-TMD, which inhibits NF-κB p65 activation by translocating into the nucleus. In the present study, we discovered that nt-p65-TMD exerted effects on surgery-induced cognitive impairment in mice. Specifically, nt-p65-TMD exhibited strong immunoregulatory properties that were able to reduce surgery-induced elevations in cerebrovascular integrity impairment, subsequent peripheral immune-cell recruitment, and inflammation amplification, which ultimately lead to cognitive decline. The nt-p65-TMD has the unique ability to regulate and reduce systemic inflammation and inflammation amplification, suggesting a new strategy for preventing development of cognitive decline that occurs in POCD.

## Introduction

Postoperative cognitive dysfunction (POCD) is a complication of peripheral surgery that is characterized by acute cognitive dysfunction, impaired memory, and loss of attention^1–4^. POCD is often associated with poor outcomes, including longer hospital stay, as well as higher mortality rates and medical costs, and imposes economic burden^4–6^. Moreover, POCD can persist over long durations, which can lead to serious central nervous system (CNS) disease^4,5,7^. Although the risk factors of POCD have been identified as infection, advanced age, and long surgery durations^1,4,7^, the precise pathophysiological mechanisms underlying POCD and optimal strategies for preventing the development of POCD remain unclear.

Episodes of POCD result from systemic and hippocampal inflammation that occurs in the clinic after surgery^3,4,8^. Activation of the immune system by surgery can trigger the infiltration of immune cells, such as macrophages and neutrophils through a disrupted blood- brain barrier (BBB), and the resulting cerebral inflammation can lead to cognitive decline^9–12^. Interestingly, the nuclear factor kappa B (NF-κB)/Rel transcription factor family has been implicated in BBB permeability and neuroinflammation, as well as in subsequent cognitive deficits^13^. This transcription factor family is considered an important regulator of inflammatory responses to injury by acting as a fuel for inflammation amplifier via a positive feedback loop^14–17^. Inhibition of NF-κB p65 subunit has been shown to suppress inflammation and modulate immune function, and specific blockade of p65 has been found to reduce intestine inflammation^18,19^. On the contrary, several studies have been demonstrated that NF-κB, which is ubiquitously localized in neurons, protects neurons against injuries and promotes cell survival^20,21^. NF-κB also exhibits protective effects on long-term potentiation (LTP), synaptic plasticity, and memory consolidation^21–23^. While NF-κB subunit c-Rel expression attenuates cell death and p50 is necessary for long term spatial memory, activation of p50/RelA (p65) dimer leads to onset of neurodegenerative disease, and p65 is associated with neuronal cell death^24–26^. Increasing evidences suggest that NF-κB inhibition improves cognitive impairment under disease conditions which inflammation is main pathologic mechanism^27–29^.

These diverse roles of NF-κB relies on complexity of NF-κB subunits^21^. Structurally, the NF-κB/Rel family consists of RelA (p65), RelB, c-Rel, NF-κB1 (p105/p50), and NF-κB2 (p100/p52), which exist as homo- or heterodimers^14,15,30^. These contain a DNA-binding (transcriptional activation) NF-κB/Rel/dorsal region sharing the N-terminal Rel-homology domain (RHD)^14,15,30^. The classical and ubiquitous form of NF-κB is a p50/p65 heterodimer, which contains the transcriptional activation domain in p65 and markedly expresses NF-kB-dependent genes^31–33^. The p65 subunit is a strong initiator of transcription^34^. The loss of p65 phosphorylation abolishes its DNA-binding activity^32,33^. Inhibitory IκB binds to NF-κB as an inactive cytoplasmic complex and controls the DNA-binding activity of NF-κB by translocating it to the nucleus^32,35^. However, translocated nuclear NF-κB participates in the activation of immune cells and controls genes that are associated with inflammatory cytokines, chemokines, cell adhesion molecules, and major histocompatibility complex proteins^14,15,36^.

In the present study, we introduce a new chemical conjugated form of NF-κB subunit p65 that contains cell-permeable peptides, also known as protein transduction domains (PTD), as a potential treatment for POCD. Choi *et al*. identified PTD from the human transcriptional factor Hph-1 and proved that PTD, Hph-1, and Hph-1-PTD can efficiently deliver therapeutic protein^37^. The PTDs are critical because they can translocate into cells, allowing them to transport other large molecules into the cells. A previous study has revealed that NF-κB attached Hph-1-PTD (nt-p65-TMD) could easily be delivered into cells and tissues, allowing it to directly target endogenous p65 in an interactomic inhibitory manner without inducing cytotoxicity^18^. Meanwhile, several studies have reported that surgery induces cognitive dysfunction *in vivo*, and patients in the POCD group show atrophy of the hippocampus, which is important to learning and memory, on MRI measurement in clinic^38–40^. Therefore, we set out to apply several behavioral methods to characterize memory after abdominal surgery in mice. The main objective of this study was to investigate the pathogenesis of surgery-induced cognitive decline and evaluate the therapeutic efficacy of nt-p65-TMD in a mouse model of POCD.

## Results

### nt-p65-TMD reduces surgery-induced memory deficits in mice

**Figure 1:**
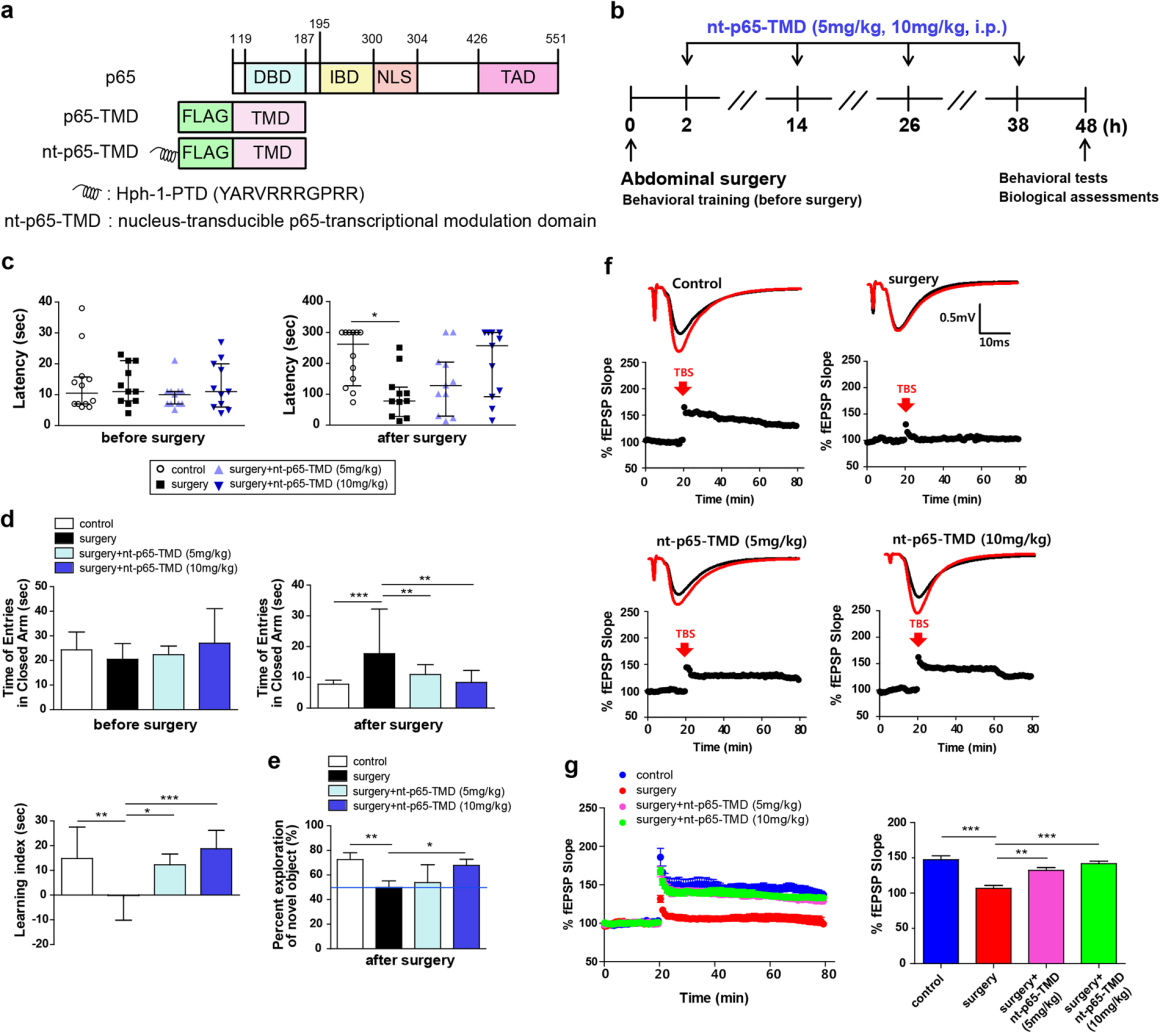
Treatment with nt-p65-TMD reduces surgery-induced cognitive dysfunction. (**a**) Construct of nt-p65-TMD. (**b**) Experimental procedures. Cognitive function was measured with the (**c**) passive avoidance test (n = 11–12), (**d**) elevated plus maze (n = 9–10), and (**e**) novel object recognition test before and 2 days after surgery (n = 6). (**f**) Representative traces and (**g**) time course of fEPSP slopes (%) from surgery mice with or without nt-p65-TMD (n = 6 slices from 3). A theta burst stimulation (TBS) protocol was delivered during the experiment. Values in behavioral tests are reported as medians and interquartile ranges, and those in electrophysiological experiments are means ± SEM. *P* values in passive avoidance test were calculated by non-parametric Kruskal-Wallis with Dunn’s multiple comparisons. *P* values in the elevated plus maze, novel object recognition test, and electrophysiological experiments were calculated with multiple comparisons by Bonferroni tests. *p < 0.05, **p < 0.01, ***p < 0.001. Statistical parameter (Supplementary Table 2).

The N-terminus of p65 has TMD-containing, DNA-binding amino acid residues and isotype-specific sequences that may participate in the functional specificity of p65^18^. To regulate p65-mediated NF-κB function *in vitro* and *in vivo*, we developed a fusion protein, which efficiently delivers p65-TMD into the nucleus of the cells, namely by fusing p65-TMD and Hph-1-PTD. The penetrating nt-p65-TMD competitively impeded the transcription of endogenous p65 at the promoter region of p65 target genes (Fig. 1a). Next, we examined whether surgery-induced POCD-like impairments could be reduced by nt-p65-TMD in mice. To determine this, mice performed several behavioral tests, including the passive avoidance test, elevated plus maze, and novel object recognition test, before and after abdominal surgery (Fig. 1b). In the passive avoidance test, all of the mice showed similar baseline latency times before surgery; however, surgery-induced reduction of latency times (memory deficits) was observed 2 days after surgery. Treatment with nt-p65-TMD (10 mg/kg) did not differ from the control groups (Fig. 1c). In the elevated plus maze test, the time to enter the closed arm before surgery did not differ among the groups in the training phase. Compared to controls, mice in the surgery group on postoperative day 2 showed longer times to enter the closed arms, while mice treated with nt-p65-TMD (10 mg/kg) showed shorter times to enter the closed arms in the test phase. Therefore, learning index was significantly greater in the surgery + nt-p65-TMD (10 mg/kg) group, compared with the surgery group (Fig. 1d, right panel). To confirm the presence of surgery-induced deficits in hippocampal-dependent learning and memory, the mice performed a novel object recognition test. Mice in the surgery group exhibited decreased discrimination of novel object and displayed no preferences, compared to the control group; however, mice in the surgery + nt-p65-TMD (10 mg/kg) group spent more time exploring the novel object than the familiar object despite the surgery, and a greater length of time exploring the novel objects, compared to the surgery group (Fig. 1e). Collectively, these results implied that the abdominal surgery induced cognitive dysfunction in mice and that nt-p65-TMD was able to inhibit surgery-induced cognitive decline.

### nt-p65-TMD reduces surgery-induced LTP deficits

Given that LTP is a putative functional correlate of learning and memory, we postulated that the administration of nt-p65-TMD would enhance LTP (synaptic efficacy) in the hippocampal region after surgery. We evoked excitatory postsynaptic potentials by electrical stimulation of the collateral Schaffer fibers, and field excitatory postsynaptic potentials (fEPSP) recordings were obtained from the stratum radiatum of the hippocampal CA1 regions. After recording baseline transmissions, we stimulated LTP through postsynaptic depolarization with tetanic stimulation. We found that the surgery group showed diminished Schaffer collateral-evoked CA1 spikes and damaged plasticity at the Schaffer collateral-CA synapses throughout the entire course of the recording, compared to other groups (Fig. 1f). However, significantly greater CA1 spikes and plasticity were exhibited in the group treated with nt-p65-TMD in terms of % fEPSP ratio, compared to the surgery group (Fig. 1g). Our electrophysiological data indicated that the LTP deficits were reduced by nt-p65-TMD, and these findings were consistent with our behavioral data. Hence, our data suggest that nt-p65-TMD enhanced synaptic strength, despite the surgery-induced synaptic impairments.

### nt-p65-TMD blocks BBB leakage and neutrophil penetration

**Figure 2:**
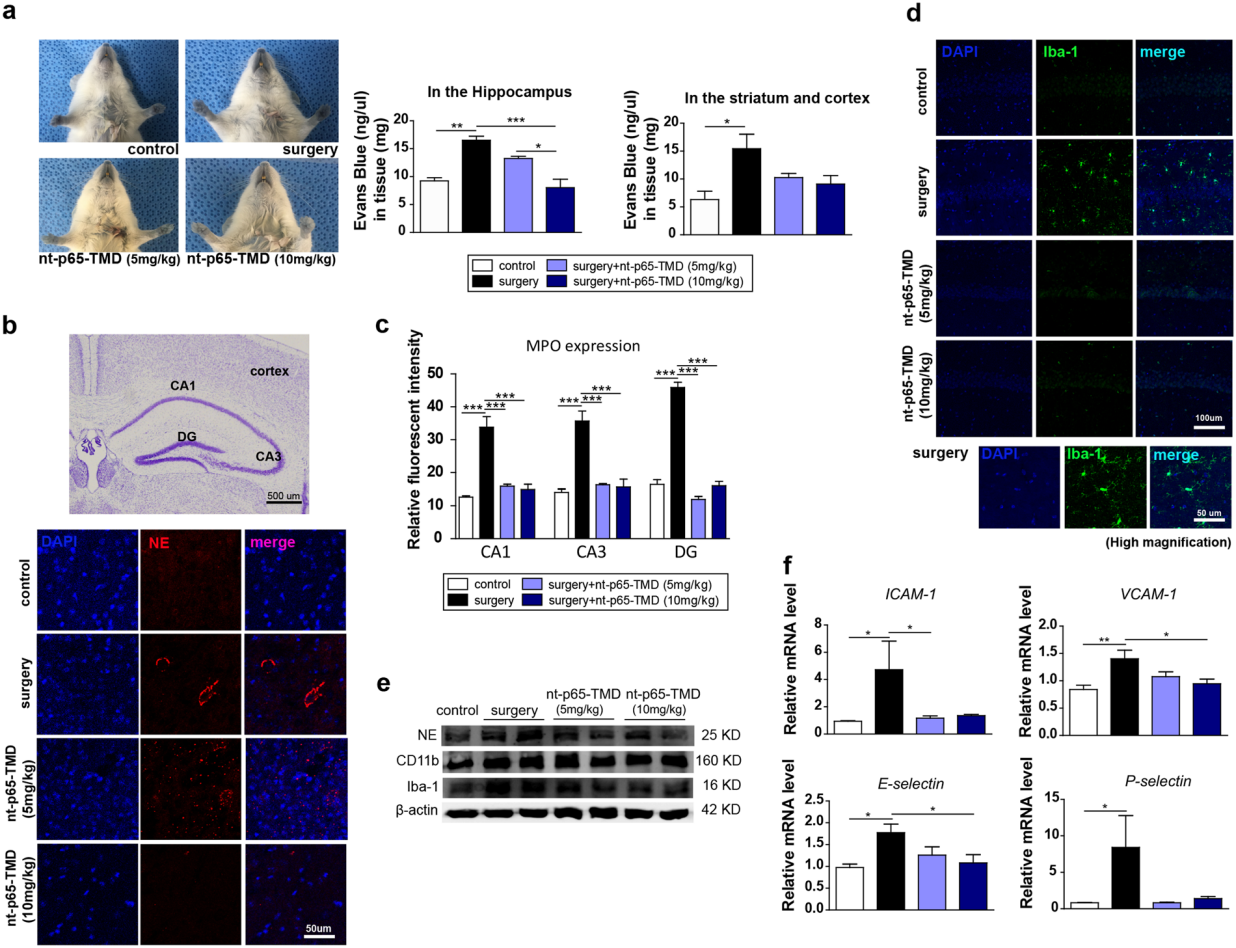
Treatment with nt-p65-TMD blocks BBB disruption and immune-cell infiltration. (**a**) BBB breakdown was assessed with Evans blue after surgery (n = 3–4). (**b**) Cresyl violet staining was performed to present anatomical areas, and immunofluorescent NE staining in the cortex was performed at 2 days after surgery. (**c**) Representative graph shows the relative fluorescent intensity for MPO in the hippocampus (n = 3–4). (**d**) Immunofluorescence staining revealed Iba-1 expression in the hippocampus. (**e**) The protein levels of NE, CD11b, and Iba-1 in the hippocampus were measured by western blotting. (**f**) The mRNA levels of *ICAM-1* (n = 7–10), *VCAM-1* (n = 6–9), *E-selectin* (n = 6–9) and *P-selectin* (n = 6–11) in the hippocampus were calculated by real-time PCR. The gels/blots are cropped from different gels and exposures. Values are means ± SEM. *P* values were calculated with multiple comparisons by Bonferroni tests. *p < 0.05, **p < 0.01, ***p < 0.001. Statistical parameter (Supplementary Table 3).

**Figure 3:**
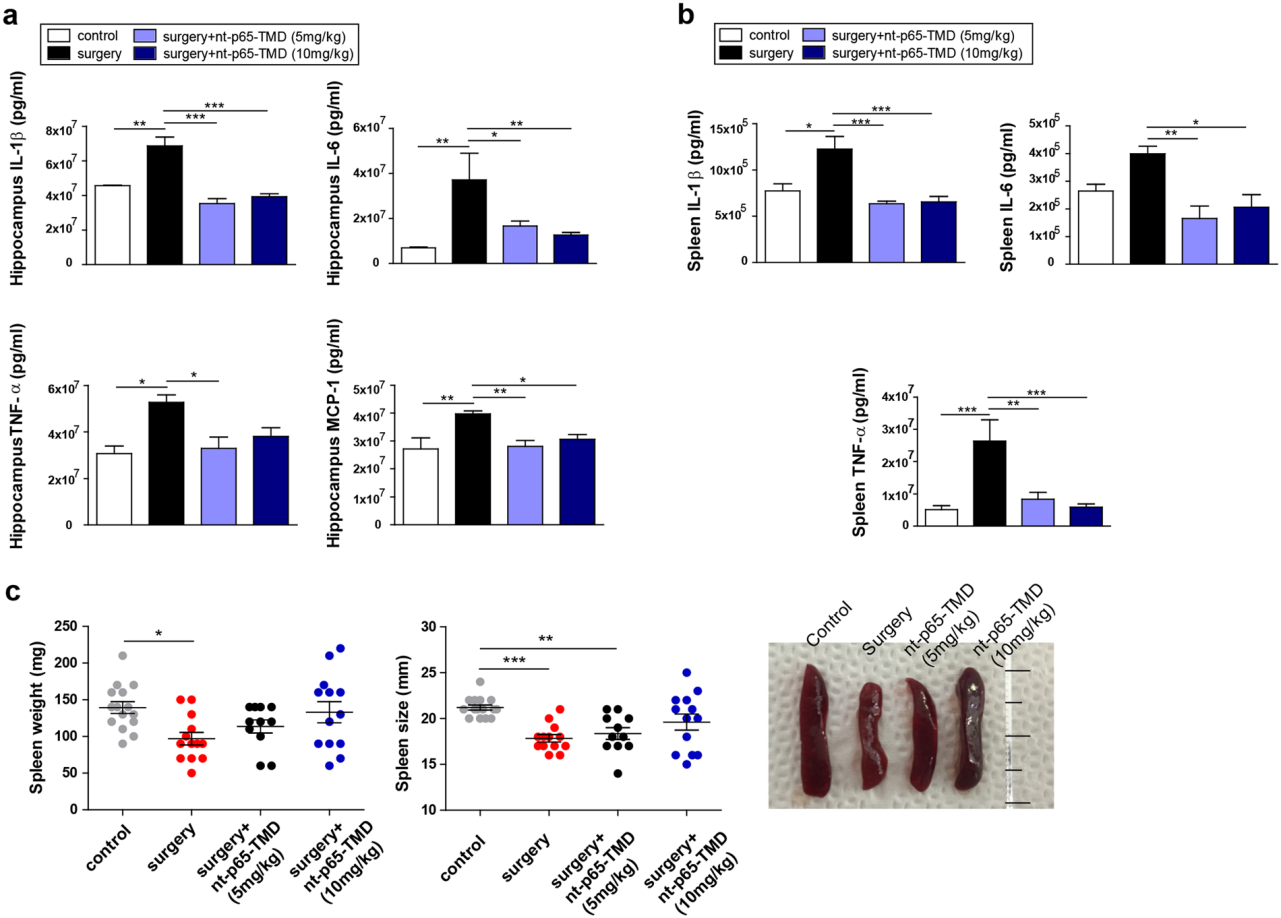
Treatment with nt-p65-TMD reduces central and systemic inflammation. At 2 days after surgery, (**a**) the hippocampal concentrations of IL-1β (n = 3–7), IL-6 (n = 3–6), TNF-α (n = 3–6), and MCP-1 (n = 4–6) and (**b**) spleen concentrations of IL-1β (n = 3–7), IL-6 (n = 3–5), and TNF-α (n = 5–8) were assessed with ELISA. (**c**) Spleen weight and size were measured during surgical intervention (n = 11–15). Values are means ± SEM. *P* values were calculated with multiple comparisons by Bonferroni tests. *p < 0.05, **p < 0.01, ***p < 0.001. Statistical parameter (Supplementary Table 4).

One of the main causes of immune-cell recruitment is BBB disruption; therefore, we characterized BBB alteration with Evans blue, a marker of albumin extravasation, and estimated BBB permeability^41^. Evans blue was successfully injected via the jugular vein, and Evans blue leakage in both brain hemispheres and the hippocampus was higher in the surgery group than it was in the control group. However, the surgery + nt-p65-TMD (10 mg/kg) group had significantly less leakage than the surgery group in the hippocampus (Fig. 2a). These structural data suggest that the BBB breakdown as a result of surgery might increase neutrophil infiltration into the brain. It is known that neutrophil elastase (NE) and his-myeloperoxidase (MPO) are necessary to the formation of neutrophil extracellular traps (NETs), thus NE and MPO are abundantly found in NETs^11^. We stained the mouse brain sections with NE and MPO to evaluate the effects of nt-p65-TMD on neutrophil extravasation. Also, western blot analysis for NE in the hippocampus was performed at 2 days after surgery (Fig. 2e). The surgery + nt-p65-TMD group showed lower levels of NE and MPO, compared to the surgery group (Fig. 2b,c,e, Supplementary Figs S1a,S1b and S2a). Inflammatory factors released from glial cells can activate the endothelium, thereby upregulating adhesion molecules that mediate leukocyte-endothelial interactions in acute inflammation. Moreover, we measured the monocyte chemoattractant protein-1 (MCP-1/CCL2) level, which is known to attract macrophage infiltration^42^. MCP-1 was elicited by surgery in the hippocampus; however, nt-p65-TMD markedly eliminated MCP-1 levels, compared to the surgery + nt-p65-TMD treated groups (Fig. 3a). To demonstrate central inflammation, we examined the expression levels of Iba-1 and CD11b, markers of microglia and macrophages, in hippocampal regions. The upregulated Iba-1 and CD11b expression induced by surgery was reduced in the group treated with nt-p65-TMD in the hippocampus (Fig. 2d and e, Supplementary Fig. S1b and S1c). The levels of adhesion molecules, such as E-selectin, P-selectin, intercellular adhesion molecule-1 (ICAM-1), and vascular cell adhesion molecule-1 (VCAM-1), were also evaluated to elucidate the effects of nt-p65-TMD on endothelium activation in the hippocampus by real-time PCR and immunohistochemistry. ICAM-1, VCAM-1, E-selectin, and P-selectin showed lower mRNA levels (Fig. 2f) and protein levels (Supplementary Fig. S1e) in the nt-p65-TMD-treated mice, compared to mice in the surgery group. These results suggest that nt-p65-TMD treatment reduced BBB permeability, along with the subsequent neutrophil infiltration and endothelial activation after surgery.

### nt-p65-TMD alleviates amplification of surgery-induced inflammation in mice

To examine the therapeutic effects of nt-p65-TMD on surgery-induced systemic and hippocampal inflammation, we intraperitoneally injected the mice with nt-p65-TMD (5 mg/kg or 10 mg/kg) at 2, 14, 26, and 38 h after surgery. Our physiological data revealed reduced spleen size and weight in the surgery group, except for the surgery + nt-p65-TMD (10 mg/kg) group (Fig. 3c). Next, we measured the levels of inflammatory cytokines in the hippocampus and spleen after the mice had performed the behavioral tests. In the spleen and hippocampus, the levels of interleukin-1 beta (IL-1β), IL-6, and tumor necrosis factor-alpha (TNF-α) were higher in the surgery group than they were in the nt-p65-TMD groups and control group. Mice treated with nt-p65-TMD had efficiently lower levels of IL-1β, IL-6, and TNF-α than mice in the surgery group (Fig. 3a and b). Concentrations of the inflammatory cytokines IL-1β, IL-6, and TNF-α in the spleen indicated the presence of a systemic inflammatory response after the surgical interventions. These data suggested that nt-p65-TMD might play a regulatory role in the host’s immune system and that nt-p65-TMD may exert its anti-inflammatory properties by suppressing pro-inflammatory cytokine secretion.

### nt-p65-TMD alters the surgery-induced inflammatory environment in the spleen and hippocampus

**Figure 4:**
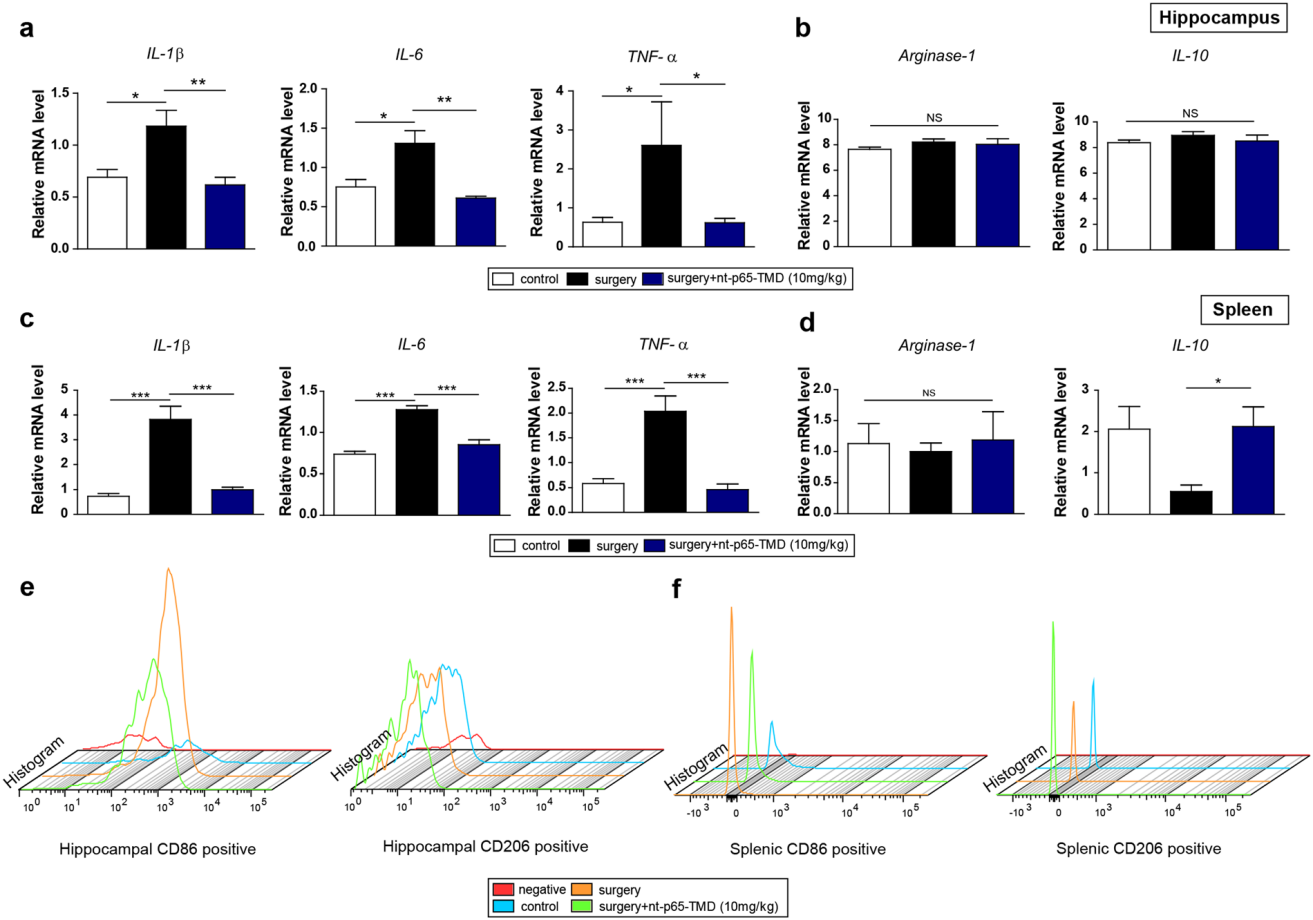
Treatment with nt-p65-TMD peripherally suppresses inflammatory mediators and enhances anti-inflammatory mediators. At 2 days after surgery, (**a**) pro-inflammatory mediator genes such as *IL-1β* (n = 9–10), *IL-6* (n = 4–5), and *TNF-α* (n = 11–16), and (**b**) anti-inflammatory mediator genes such as *arginase-1* (n = 5) and *IL-10* (n = 5) in the hippocampus and (**c**) pro-inflammatory mediator genes, such as *IL-1β* (n = 12), *IL-6* (n = 12), and *TNF-α* (n = 9–10) and (**d**) anti-inflammatory mediator genes, such as *arginase-1* (n = 12) and *IL-10* (n = 12) in the spleen were measured with real-time PCR. (e and f) Flow cytometry was performed with tissue from the hippocampus and spleen pro-inflammatory cell surface marker: CD86, anti-inflammatory cell surface marker: CD206 among CD11b^+^ and CD45^+^ cells). Histogram shows pro- or anti-inflammatory positive cells in the hippocampus (**e**) and spleen (**f**). Values are means ± SEM. *P* values were calculated with multiple comparisons by Bonferroni tests. *p < 0.05, **p < 0.01, ***p < 0.001. Statistical parameter (Supplementary Table 5).

Next, we investigated the effects of nt-p65-TMD on the gene expression levels of pro-inflammatory mediators (*IL-1β*, *IL-6*, and *TNF-α*) and anti-inflammatory mediators (*arginase-1* and *IL-10*) in the hippocampus and spleen. In the hippocampus, surgery elicited pronounced pro-inflammatory-associated gene expression (Fig. 4a). Treatment with nt-p65-TMD (10 mg/kg) reduced *IL-1β*, *IL-6*, and *TNF-α* expression, with no changes in *arginase-*1 or *IL-10* expression on postoperative day 2 in the hippocampus (Fig. 4a and b). Surgery significantly increased the mRNA levels of pro-inflammatory-associated genes, such as *IL-1β*, *IL-6*, and *TNF-α*, in the spleen, compared to the control group; however, treatment with nt-p65-TMD reduced expression of these genes, compared to the surgery group (Fig. 4c). We noted a tendency for higher *IL-10* mRNA expression in nt-p65-TMD-treated mice, while no difference in *arginase-1* was noted among all groups (Fig. 4d). To directly investigate whether nt-p65-TMD treatment would regulate the gene expression of pro- or anti- inflammatory mediators in microglia and peripheral macrophages, we performed flow cytometry. We separated the resident and blood-derived immune cells expressing the specific surface antigens CD11b and CD45 from other cells within the hippocampus and spleen. Primary gating was based on forward and side light scatter, respectively, and the merged histograms indicated that nt-p65-TMD (10 mg/kg) treatment reduced the amount of CD86^+^ cells among CD11b^+^/CD45^+^ cells in the hippocampus and spleen compared to the surgery group, and elevated the amount of CD206^+^ cells among CD11b^+^/CD45^+^ cells in the spleen, compared to the other groups (Fig. 4e and f).

### nt-p65-TMD alleviates LPS-induced cognitive dysfunction and inflammation

**Figure 5:**
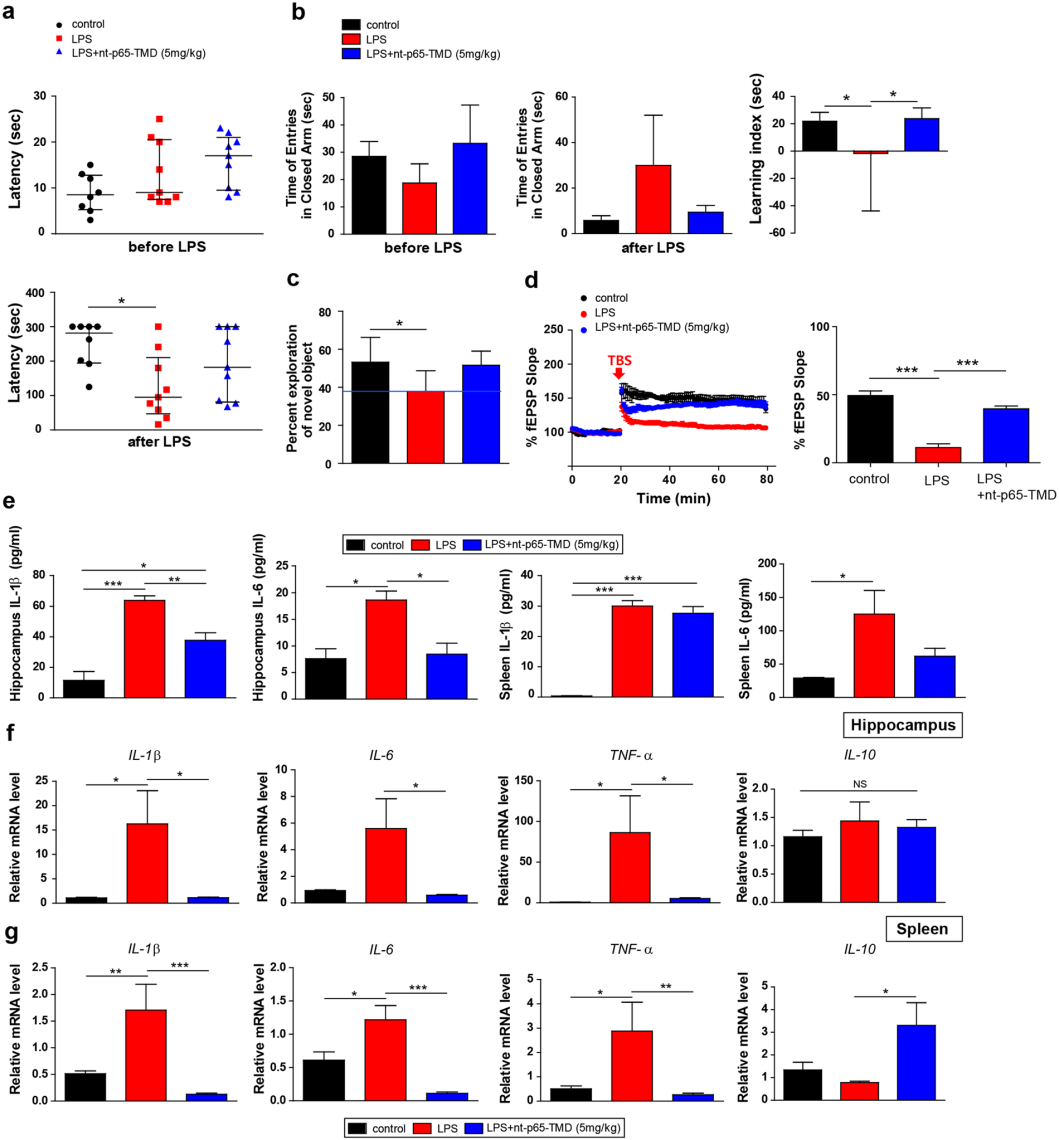
Treatment with nt-p65-TMD inhibits systemic inflammatory challenge-induced cognitive dysfunction and inflammation amplification. At 2 days after LPS challenge, cognitive function was evaluated with the (**a**) passive avoidance test (n = 8–9), (**b**) elevated plus maze (n = 6–7), and (**c**) novel objective recognition test (n = 6). (**d** and **f**) Representative traces and (**g**) time course of fEPSP slopes (%) from LPS-treated mice with or without nt-p65-TMD. A theta burst stimulation (TBS) protocol was delivered during the experiment (n = 4–6 slices from 3). (**e**) Hippocampal IL-1β (n = 3–5) and IL-6 (n = 3–6) levels and spleen IL-1β (n = 5–9) and IL-6 (n = 4–6) levels were measured with ELISA. (**f**) Hippocampal IL-1β (n = 16–18), IL-6 (n = 13–16), TNF-α (n = 12–16), and IL-10 (n = 5) transcripts and spleen IL-1β (n = 16–18), IL-6 (n = 6–8), TNF-α (n = 7–12), and IL-10 (n = 4–5) were detected by real-time PCR at 2 days after LPS injection. Values in the behavioral tests are reported as medians and interquartile ranges, and those in the electrophysiological, ELISA, and real-time PCR experiments are presented as means ± SEM. *P* values in passive avoidance test were calculated by non-parametric Kruskal-Wallis with Dunn’s multiple comparisons. *P* values in elevated plus maze, novel object recognition test, electrophysiological experiments, ELISA, and real-time PCR were calculated with multiple comparisons by Bonferroni tests. *p < 0.05, **p < 0.01, ***p < 0.001. Statistical parameter (Supplementary Table 6).

To examine the therapeutic effects of nt-p65-TMD on systemic inflammation, we corroborated our findings in an additional independent cohort of mice that were administered LPS systemically. This systemic inflammatory challenge resulted in cognitive dysfunction, similar to the findings of the surgery model. In the passive avoidance test, the latency time (sec) to enter the dark compartment in the LPS group was shorter than times in the PBS-injected control group and LPS + nt-p65-TMD (5 mg/kg) group at 2 days after LPS administration (Fig. 5a). In the elevated plus maze, the LPS group displayed a reduced entry time to the closed arm, compared to the control, but this reduction was not observed in the LPS + nt-p65-TMD group. Therefore, the learning index in the LPS + nt-p65-TMD group was enhanced, compared with the LPS group (Fig. 5b). Furthermore, mice in the LPS group displayed a reduced percentage of novel object recognition memory, compared with the control group; however, the LPS + nt-p65-TMD group did not differ from the control group on any of the novel object recognition test parameters (Fig. 5c). Regarding LTP, compared to the control, LPS-challenged mice showed an earlier return to baseline and damaged plasticity; while the LPS + nt-p65-TMD group elicited higher fEPSP slopes, indicating an increase of efficacy in synaptic transmission, compared to the LPS group (Fig. 5d). After systemic inflammatory challenge by LPS, high levels of inflammatory mediators, including IL-1β and IL-6, and high gene expression of pro-inflammatory cytokines, such as IL-1β, IL-6 and TNF-α, were observed in the spleen and hippocampus (Fig. 5e–g). These elevated cytokine and gene expression levels identified in the LPS group were not found in mice subjected to nt-p65-TMD (5 mg/kg). In addition, the LPS + nt-p65-TMD group showed higher expression of the anti-inflammatory gene *IL-10* in the spleen, compared to the LPS group (Fig. 5e–g). These data suggest that the artificially increased systemic inflammation induced by LPS caused cognitive deficits and the release of pro-inflammatory mediators, while the systemic administration of nt-p65-TMD suppressed the LPS-induced cognitive dysfunction and inflammatory amplification.

### nt-p65-TMD suppresses inflammatory amplification in microglia and macrophages

**Figure 6:**
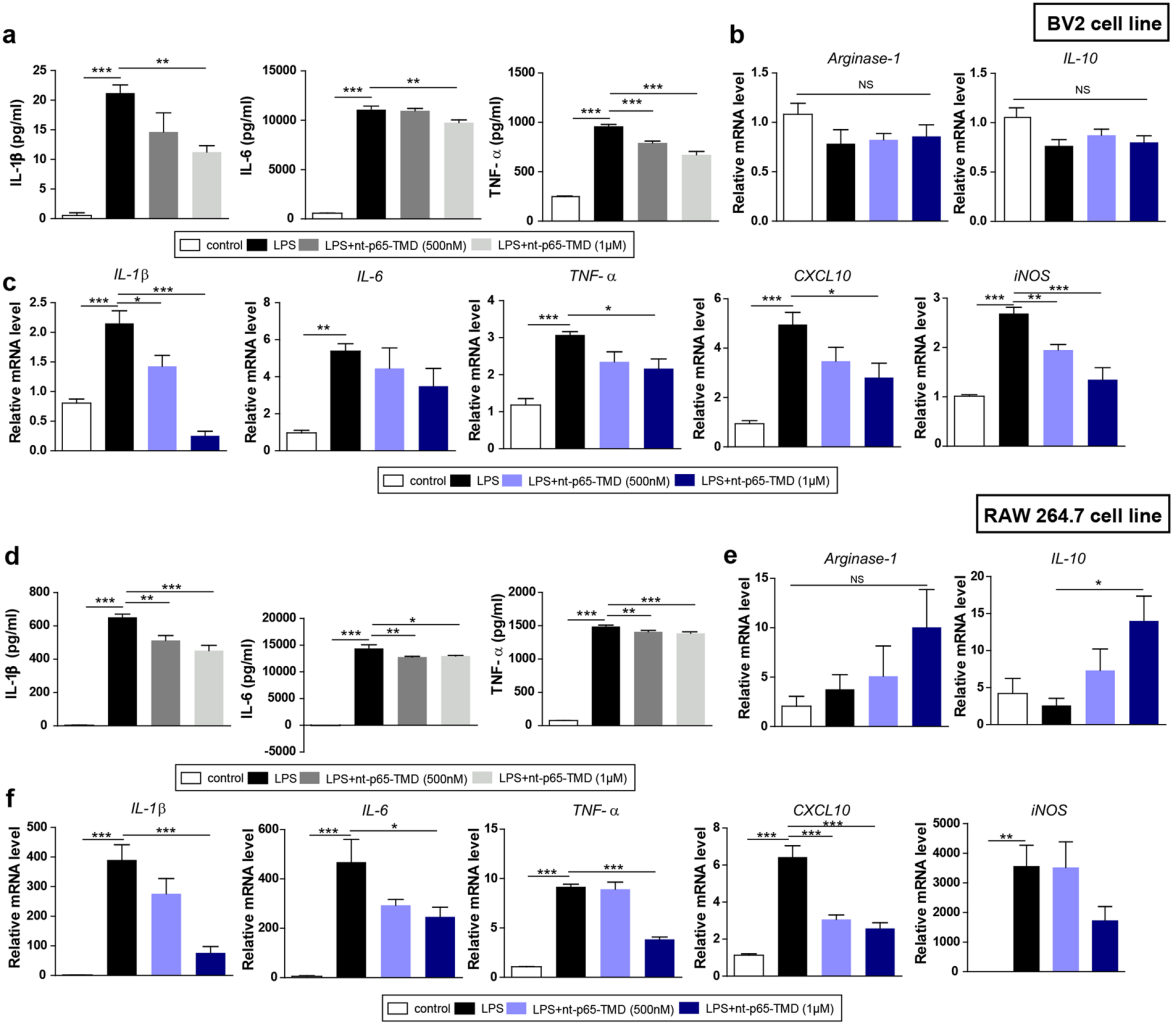
Treatment with nt-p65-TMD reduces inflammatory mediators in BV2 microglia and RAW264.7 macrophages and elevates anti-inflammatory mediators in the RAW264.7 macrophages. (**a**) The culture supernatants from the BV2 cell line were collected 24 h after LPS stimulation and the IL-1β (7 repeats), IL-6 (7 repeats), and TNF-α (7 repeats) levels were evaluated by ELISA. (**b**) The transcripts of anti-inflammatory mediator genes such as *arginase-1* (7 repeats) and *IL-10* (7 repeats) and (**c**) pro-inflammatory mediator genes such as *IL-1β* (4–5 repeats), *IL-6* (6 repeats), *TNF-α* (6–7 repeats), *CXCL10* (7 repeats), and *iNOS* (7 repeats), of BV2 microglia were measured by real-time PCR. (**d**) The culture supernatants from the RAW264.7 cell line were collected 24 h after LPS stimulation, and the IL-1β (7 repeats), IL-6 (5 repeats), and TNF-α (5 repeats) levels were assessed by ELISA. (**e**) The transcripts of anti-inflammatory mediator genes, such as *arginase-1* (5–6 repeats) and *IL-10* (5 repeats), and (**f**) pro-inflammatory mediator genes, such as *IL-1β* (5–7 repeats), *IL-6* (5 repeats), *TNF-α* (7 repeats), *CXCL10* (7 repeats), and *iNOS* (6 repeats), of RAW 264.7 macrophages were calculated by real-time PCR. Values are means ± SEM. *P* values were calculated with multiple comparisons by Bonferroni tests. *p < 0.05, **p < 0.01, ***p < 0.001 vs. LPS. Statistical parameter (Supplementary Table 7).

To further investigate the results of the *in vivo* study, we generated cell cultures of microglial and macrophage cell lines, which are associated with production of inflammation-associated molecules^43^. We speculated that nt-p65-TMD treatment would affect inflammatory cytokine secretion in microglia and macrophages. To demonstrate these effects, we performed an *in vitro* study in cultured BV2 microglia and RAW264.7 macrophages under LPS stimulation to induce inflammation. Before the induction of inflammation by LPS, nt-p65-TMD (500 nM or 1 μM) was transduced into microglia or macrophages for 2 h. When BV2 cells were treated with 1 μg/mL of LPS, microglia released pro-inflammatory cytokines including TNF-α, IL-6, and IL-1β, compared to the controls, whereas the levels of these cytokines were reduced in nt-p65-TMD (1 μM)-treated microglia (Fig. 6a). In addition, microglia stimulated with LPS expressed the genes of pro-inflammatory cytokines such as *IL-1β*, *IL-6*, *TNF-α*, *CXCL10*, and *iNOS*, although this expression was significantly inhibited by nt-p65-TMD treatment except expression of *IL-6* (Fig. 6c). However, nt-p65-TMD did not affect the elevation of anti-inflammatory mediator genes, including *IL-10* and *arginase-1*, compared to the LPS group (Fig. 6b). We also determined the level of pro-inflammatory cytokine secretion in RAW264.7 cells stimulated with LPS to further investigate the anti-inflammatory effects of nt-p65-TMD. The production of TNF-α, IL-6, and IL-1β following LPS stimulation was enhanced compared to basal levels, while nt-p65-TMD (500 nM or 1 μM) significantly attenuated their production in RAW264.7 cells (Fig. 6d). We next examined mRNA expression of pro- or anti-inflammatory mediator genes. Similar to microglia, macrophages also generated higher expression profiles for *IL-1β*, *IL-6*, *TNF-α*, *CXCL10*, and *iNOS* after LPS stimulation, compared to the control, and treatment with nt-p65-TMD (1 μM) suppressed *IL-1β*, *IL-6*, *TNF-α* and *CXCL10* from being upregulated (Fig. 6f). In macrophages, the expression of *IL-10* was significantly higher with nt-p65-TMD, compared to the LPS group, while the *arginase-1* mRNA levels were not significantly different among the groups (Fig. 6e). These data indicate that nt-p65-TMD may act as a modulator of inflammatory mediators in microglia and macrophages.

## Discussion

Currently, POCD has emerged as a major clinical problem associated with high mortality and morbidity. Therefore, indentifying new therapeutics for the prevention or treatment of POCD based on its pathological mechanisms is essential, especially since existing treatments are limited. Symptoms of POCD are closely linked to inflammation. Therefore, in the present study, we focused on NF-κB, which plays a key role in various inflammatory diseases. Specifically, we developed the fusion protein nt-p65-TMD, which includes an intranuclear transducible form of the RelA (p65) transcriptional modulation domain (TMD) and a human-origin Hph-1-PTD that allows nt-p65-TMD to be delivered efficiently into the nucleus and to inhibit p65. In our *in vivo* mouse study, nt-p65-TMD administration reduced surgery-induced cognitive decline, as measured with several behavioral tests, and reduced surgery-induced LTP damage according to electrophysiology assays. Surgery-induced peripheral immune stimulation and ensuing neuroinflammation were suppressed by nt-p65-TMD treatment according to lower levels of pro-inflammatory cytokines, including IL-1β, IL-6, and TNF-α, in the spleen and hippocampus. We also found that surgery-induced BBB breakdown and subsequent immune-cell infiltration were inhibited by nt-p65-TMD. Additionally, nt-p65-TMD attenuated the expression levels of pro-inflammatory genes in the spleen and hippocampus and selectively elicited anti-inflammatory genes in the spleen.

Our experiments with artificially upregulated systemic inflammation, as induced by LPS, revealed cognitive dysfunction, damaged LTP, and amplified inflammation in the hippocampus and spleen; however, nt-p65-TMD administration efficiently reduced these LPS-induced symptoms. We also noted that nt-p65-TMD upregulated anti-inflammatory genes systemically. In our *in vitro* study, nt-p65-TMD was applied to LPS-primed BV2 microglia and RAW264.7 macrophage cell lines. After LPS challenge, treatment with nt-p65-TMD reduced the amplified secretion of pro-inflammatory cytokines in BV2 microglia and RAW264.7 macrophages, although the upregulation of anti-inflammatory cytokines was only observed in RAW264.7 macrophages. The results of the present study help clarify the mechanisms underlying POCD as it relates to nt-p65-TMD. Specifically, we think that nt-p65-TMD likely inhibits the positive feedback loop of inflammation amplification and subsequently inhibits BBB breakdown and peripheral immune-cell infiltration stemming from systemic inflammation, which is produced by surgery or inflammatory challenge.

Inflammation is considered the main characteristic of several pathological conditions, and inflammation amplifiers are connected with various human diseases, including autoimmune, neurodegenerative, and other inflammatory diseases^12,44^. Inflammation amplifiers are activated by simultaneous NF-κB and STATs activation in non-immune cells, which are stimulated by various factors derived from immune cells, neural cells, or fibroblasts, although the main amplification signal is NF-κB^16,44^. The NF-κB pathway is mediated by IL-6 through a positive feedback loop that reactivates NF-κB signaling^16,44^. Since NF-κB exists in all cell types and regulates the activation of immune cells^15,45^, this makes it an important target for regulating inflammation amplification in inflammation-mediated diseases. It is likely that the intraperitoneal injection of nt-p65-TMD reduced inflammation amplification systemically, which caused an accumulation of activated immune cells and the release of various inflammatory mediators that opened the BBB^16^. It is known that NF-κB positively regulates IL-1β^46^, TNF-α^47^, IL-6^48^, iNOS^49^, and CXCL10^50,51^, and that the inhibition of NF-κB elicits immunosuppressive effects^52^. Immune cells that secrete IL-1β, IL-6, TNF-α, and iNOS and have the cell surface markers CD80 and CD86 have cytotoxic effects that can exacerbate tissue damage^43,53,54^. In our study, real-time PCR showed that the expression of genes that mediates inflammation, including *IL-1β, IL-6*, and *TNF-α*, in the hippocampus and spleen tissues were upregulated by surgery or LPS administration, and were suppressed by nt-p65-TMD owing to the inhibition of NF-κB. Further, flow cytometry revealed that increases in CD86^+^ cells in microglia/macrophages were reduced by nt-p65-TMD owing to the inhibition of NF-κB. Similarly, our data from single cell line cultures (BV2 microglia and RAW264.7 macrophages) showed that *IL-1β, IL-6, TNF-α, iNOS*, and *CXCL10* genes that were upregulated by LPS challenge were reduced by nt-p65-TMD treatment. In both the spleen and RAW264.7 macrophages, nt-p65-TMD treatment elicited the release of the anti-inflammatory cytokine IL-10 and elevated the number of cells with the cell surface marker CD206, which is linked to tissue repair, regeneration, and remodeling^43,54^. These results indicate that nt-p65-TMD regulates inflammation amplification and modulates systemic inflammation towards anti-inflammatory environments.

The mechanisms of neuroinflammation are closely related with the CNS and peripheral tissues, including the gastro-intestinal tract and spleen^55^. The peripheral immune system delivers inflammatory elements to the brain through the blood stream and subsequently increases BBB permeability^55,56^. The BBB is a monocellular layer that normally separates and conjoins peripheral circulation and the CNS^55–57^. However, in pathologic conditions, the BBB is disrupted by LPS or inflammatory cytokines, which leads to cytokine transport and immune-cell infiltration into the brain^56,57^. Neutrophils, which are circulating leukocytes, are captured, slowly rolled, and then transferred into the BBB by interaction among vascular endothelial surface molecules^58^. Selectins, a family of cell adhesion molecules, participate in leukocyte rolling, and both VCAM-1 and ICAM-1 bind to β1 and β2 integrins on the leukocyte surface during the arresting step, allowing leukocytes to enter into inflamed areas^58,59^. Recruited neutrophils produce NETs composed of NE and MPO^11,60^. Peripheral macrophages are also recruited through BBB leakage and play a central role in regulating the inflammatory response to pathologic conditions by influencing the injury or repair processes^61^. Excessive infiltration of neutrophils and macrophages (immune cells) can exacerbate brain damage by producing inflammatory mediators, including IL-1β, IL-6, and TNF-α^58^. Microglia are the immune cells of the CNS and are distributed throughout the brain in order to monitor the environment^61,62^. Microglia activated in response to pathologic stimuli also secret the inflammatory cytokines IL-1β, IL-6, and TNF-α and produce neurotoxic reactive oxygen species^63^. In the present study, surgery elicited dysfunction of the BBB and elevated the expression of several vascular inflammatory molecules, including P-selectin, E-selectin, VCAM-1, and ICAM-1, in the hippocampus. Neutrophil-associated factors, such as NE and MPO, were found in the hippocampus or cortex of mice in the surgery group. In addition, the microglia and macrophage markers Iba-1 and CD11b were highly expressed in hippocampal regions in response to surgery. Also, pro-inflammatory cytokines, including IL-1β, IL-6, and TNF-α, were increased in the hippocampus after surgery/LPS challenge. These surgery-induced outcomes were reduced by treatment of nt-p65-TMD. The previous studies support out data that NF-κB plays a central role in immune-cell activation by controlling gene expression as a promoter or enhancer through cytokines (IL-1, IL-6, TNF-α and chemotactic protein), cell adhesion molecules (V-CAM-1, ICAM-1, and E-selectin), cytokine receptors, and growth factors (G-CSF and M-CSF) after infection or injury^15,45,64^. Under stress conditions, the spleen is responsible for initiating immune reactions, and splenic contraction is easily observed following acute injury^65^. Also, surgical stress led to splenic contraction^65^. Our *in vivo* data showed that surgery elicited upregulation of inflammatory mediators in the spleen, which is a peripheral lymphoid organ that regulates the immune response. Meanwhile, blockade of NF-κB prevented not only splenic inflammation, but also BBB breakdown, and activation of brain-penetrant and -resident immune cells with commensurate reductions in inflammatory amplification after surgery.

Inflammatory cascades are believed to amplify neuroinflammation and to impair synaptic plasticity, which is required for learning and memory^9–11^. Our data showed that augmented inflammatory mediators were expressed in the hippocampus after surgery/LPS injection, suggesting that an inflamed hippocampus might result in cognitive decline. Since NF-κB signaling is also stimulated by peripheral surgery and is crucial in cognitive impairment^18,27,66^, we anticipated that NF-κB would play a negative role in cognitive function. As we expected, mice after surgery showed poor functional outcomes on hippocampal-dependent behavioral tests (passive avoidance test, elevated plus maze, and novel object recognition test) and had damaged LTP in the electrophysiological tests. However, these surgical events were prevented by nt-p65-TMD administration. Nonetheless, several studies have demonstrated that neuronal NF-κB participates in learning and memory that a subunit of NF-κB strengthens long term memory and synaptic plasticity^21–23,26^. The dual role of NF-κB is likely dependent on cell types and pathologic characteristics. Inhibition of NF-κB in pathologic conditions, wherein inflammation is the primary mechanism, has been found to improve cognitive dysfunction^27–29^.

During our experiments, we encountered several intriguing questions. First, some of the *in vivo* data did not show a dose-dependent relationship, compared to *in vitro* data. Mice treated with a smaller concentration of nt-p65-TMD (5 mg/kg), compared to those treated at a larger dose of nt-p65-TMD (10 mg/kg), showed lower in hippocampal cytokine levels such as TNF-α and MCP-1 by ELISA. It is likely that nt-p65-TMD exerts systemic protection, therefore, in the hippocampus, nt-p65-TMD showed dose-independent protection. Another question was whether nt-p65-TMD is directly introduced into the brain and controls cognitive function. In our pilot study, nt-p65-TMD was not detected in the hippocampus of the brain until the second postoperative day (data not shown). We believe that nt-p65-TMD systemically reduced inflammation and thus prevented cognitive dysfunction. However, when nt-p65-TMD was directly applied via intracerebrovascular injection, surgery-induced cognitive dysfunction was also improved in our pilot study (data not shown). Thus we needed to confirm the role of NF-κB in diseases where inflammation was the main pathological factor. This investigation is our follow-up study. Collectively, blockade of NF-κB prevented impairment of memory and synaptic plasticity, inhibiting peripheral inflammation after surgery.

In conclusion, our findings demonstrate a relationship between NF-κB and systemic inflammation in POCD. Furthermore, our data suggest that systemic nucleus-transducible NF-κB antagonism has the potential to indirectly reduce cognitive and synaptic-plasticity dysfunctions by normalizing BBB integrity and avoiding immune-cell recruitment during inflammatory challenges. The nt-p65-TMD also acts as a potent regulator of inflammation amplification, which may provide a novel basis for research into the mechanism of POCD and cognitive decline repair. Taken together, our results implied that modulating systemic inflammatory amplification with nt-p65-TMD will be of great significance in the treatment of POCD.

## Methods

### Ethics statement

All experimental procedures in this study were approved by the Institutional Animal Care and Use Committee of Yonsei University Health System, which is certified by the Association for Assessment and Accreditation of Laboratory Animal Care International. Animal experiments were performed in accordance with the Guide for the Care and Use of Laboratory Animals issued by the Institute of Laboratory Animal Resources Commission on Life Science National Research Council, USA.

### Preparation of the nucleus-transducible form of nt-p65-TMD

The FLAG-tagged p65-DBD (p65-TMD) was encoded with amino acids (1–187) and conjugated with Hph-1-PTD. The fusion proteins were amplified using polymerase chain reaction (PCR) from the full-length mouse p65 (1–551) and inserted into the pET-28a ( + ) vector (Novagen, Madison, WI, USA) as described in a previous study^18^. Using commercial fluorenylmethoxycarbonyl amino acids (Novabiochem, Darmstadt, Germany), the Hph-1-PTD (YARVRRRGPRR-OH) was prepared with the solid-phase synthesis method on an Applied Biosystems 433 A peptide synthesizer (Thermo Fisher Scientific, Waltham, MA, USA). The entire conjugate was produced by sequentially linking the target protein to the N-terminal end of the synthesized PTD via peptide bonds.

### Animal models

Male CrljOri:CD1 (ICR) mice were used in this study (OrientBio, Seongnam, Gyeonggi-Do, Korea; aged 8–12 weeks, Swiss mice from non-inbred stock (seven female and two male albino) from Dr. de Coulon’s laboratory, Switzerland. All mice were housed in temperature and humidity controlled pathogen-free facilities with 12-h light-dark cycles at Yonsei University College of Medicine. Mice were maintained in groups of five per cage with free access to food and water.

### Abdominal surgery model

Mice were anesthetized by 3% sevoflurane in mixed gas, and anesthesia was maintained with 2% sevoflurane for 2 h. Mice were placed onto a homeothermic blanket control unit and their rectal temperature was maintained at 37 ± 0.5 °C. Once the mice were sufficiently sedated, the skin of the abdomen was sterilized with 70% ethanol. For abdominal surgery, the intestines were exposed through a 2-cm midline incision and rubbed for 30 s; this was repeated four more times for a total of five trials. The interval between each trial was 30 s. In addition, the superior mesenteric artery was clipped twice for 20 min, with the clippings separated by 10 min interval. After the mice had been under general anesthesia for 2 h, the wound was closed using sterile silk sutures. Mice were administered buprenorphine 0.05 mg/kg subcutaneously and returned to their home cages, where they were monitored for 2 days postoperatively. The nt-p65-TMD was injected intraperitoneally at 2, 14, 26, and 38 h after surgery. The *in vivo* abdominal surgery experimental group was divided into four subgroups as follows: (1) normal control group (control), (2) surgery group, (3) surgery + nt-p65-TMD (5 mg/kg) group, and (4) surgery + nt-p65-TMD (10 mg/kg) group. Mice were randomly placed into the above groups to reduce bias.

### LPS model

To induce systemic inflammation, LPS (*Escherichia coli* serotype 055:B5) was purchased from Sigma-Aldrich (St. Louis, MO, USA), diluted with phosphate-buffered saline (PBS), and administered intraperitoneally at a dose of 20 mg/kg in accordance with a previous study^18^. The nt-p65-TMD was injected intraperitoneally at 2 and 14 h after LPS treatment. Intraperitoneally PBS-injected mice were used as the control. Afterward, mice were returned to their cages and monitored for the next 2 days. The *in vivo* LPS experimental group was divided into three subgroups as follows: (1) normal control group (control), (2) LPS (20 mg/kg) group, and (3) LPS (20 mg/kg) + nt-p65-TMD (5 mg/kg) group. Mice were grouped randomly to reduce bias.

### Behavioral tests

The passive avoidance test and elevated plus maze were performed before modeling (abdominal surgery or LPS) and 2 days after modeling. In the case of the novel object recognition test, mice underwent habituation period for 2 days before modeling. Mice were sacrificed after the behavioral tests.

### Passive avoidance test

The passive avoidance test is used to evaluate learning and memory and is based on the tendency of mice to show a fear response (electric foot shock). This test was performed in a 41 × 21 × 30 cm plastic chamber consisting of light and dark compartment that were separated by a guillotine door (JEUNGDO Bio & Plant Co., Ltd., Seoul, Korea). Stainless steel bars on the floor of the dark compartment were capable of administering an electric shock. Each mouse was to undergo one training trial and one test trial. In the training trial before modeling, one mouse was placed in the light compartment and allowed to explore the chamber for 10 s, after which guillotine door between the light and dark sides was opened. When the mouse entered the dark sides, the guillotine door was closed and an electric foot shock (0.5 mA) was administered for 3 s. The time it took the mouse to enter the dark side (latency time) was recorded. The mouse was then transferred to its home cage. To test learning and memory in the test trial, on postoperative or post-LPS-injection day 2, the mouse was place back in the conditioning light chamber. The time to enter the dark compartment was immediately recorded. The maximum time of passive avoidance task was 5 minutes without electric shock. After every trial, the apparatus was cleaned with 70% ethanol. The mice were sacrificed after the tests.

### Elevated plus maze

The elevated plus maze consisted of two open arms (31 × 6 × 1 cm) and two enclosed arms (31 × 6 × 15 cm) with a central open square area (5 × 5 × 1 cm) (JEUNGDO Bio & Plant Co., Ltd.). Each arm in the maze was at a 90° angle, and the maze was raised 40 cm above the floor. In the training phase, each mouse was placed at the end of the open arm facing the other open arm and allowed to explore freely for 5 min. The time it took the mouse to first enter the closed arms was recorded by an overhead video-scanning system. To test their learning and memory, on postoperative or post-LPS-injection day 2, in the test phase, the elevated plus maze was performed again. The latency time to enter the closed arms was recorded, and learning indices were presented as graphs (latency time of training period – latency time of test period = learning index). After every trial, the apparatus was cleaned with 70% ethanol. The behavioral data were analyzed using a SMART Video Tracking system (Panlab Harvard Apparatus, Barcelona, Spain) with SMART v2.5.21 software.

### Novel object recognition test

Hippocampus-dependent visual and spatial short term memory was evaluated with the novel object recognition test^67^, which was performed in a black rectangular box (40 × 40 × 40 cm) box with an open top (JEUNGDO Bio & Plant Co., Ltd.). Each mouse was habituated to the box in the absence of objects for 5 min for two consecutive days. In the familiarization phase, an individual mouse was presented with two identical objects to familiarize and allowed to explore freely for 3 min. The latency time to explore the two objects was recorded by a computer equipped with a video scanning system. To test the mouse’s learning and memory, on postoperative or post-LPS-injection day 2, in the test phase, one of the familiar objects was replaced with a novel object. After then, the mouse was introduced into the testing box again and allowed to move freely for 3 min. The time to explore and stay near the familiar or novel object was measured by a computer-based video scanning system. The behavioral data were analyzed with the SMART v2.5.21 software. Recognition memory was expressed as a percentage of novel object preference, which was calculated as the time spent exploring either of the two identical objects in the familiarization phase or the novel object in the test phase during total exploration time.

### Long-term potentiation

Two days after surgery, mice were anesthetized with 2% isoflurane. The brains were isolated and subsequently cooled by cardiac perfusion with cold sucrose artificial cerebrospinal fluid (aCSF; 195.5 mM sucrose, 2.5 mM KCl, 2.5 mM CaCl_2_, 1.3 mM MgSO_4_, 1 mM NaH_2_PO_4_, 26.2 mM NaHCO_3_, 11 mM glucose, 2 mM Na pyruvate, and 1 mM Na ascorbate, supplemented with 95% O_2_/5% CO_2_). Coronally sectioned brain slices (400-μm thick) were incubated with aCSF at 35 °C for 30 min to recover, and then incubated at room temperature for 1–4 hours before recording. Slices were transferred to a submersion chamber. In the electrophysiological experiments, the hippocampal CA3 and dentate gyrus areas were cut away to isolate the CA1 area before starting the LTP measurements. Electrodes (3–6 MΩ resistance) were used to obtain fEPSP in the CA1 stratum radiatum; specifically, fEPSPs were evoked by Schaffer collateral (SC)/commissural fibers with a concentric bipolar electrode placed 200–300 μm from the recording pipette. A 20-min baseline recording at 0.1 Hz with a 0.2-ms pulse duration was obtained before producing theta burst stimulation (TBS) protocol (4 pulses at 100 Hz) separated by a 20-s inter-train interval to induce LTP. A voltage was applied at 50% of the maximum fEPSP for LTP. Under visual guidance using infrared (IR) differential interference contrast (DIC) microscopy, whole-cell recordings were obtained from neurons. Input/output (I/O) curves were obtained by plotting the slope of the recorded fEPSPs after stimulating the slice with increasing voltages (0.3–0.9 V). The field potential was recorded every 30 s for 2 h using an axopatch 1D amplifier (Molecular Devices, Sunnyvale, CA, USA) digitized at 10 kHz and filtered at 2 kHz with the Digidata 1322 A acquisition system and AXON™ pClamp 9.0 software (Molecular Devices). These experiments were performed at 27–29 °C.

### BBB extravasation

BBB disruption was investigated by Evans blue extravasation methods. Evans blue was diluted in PBS (0.5%) and filtered to remove particles and air bubbles. On postoperative or post-LPS-injection day 2, mice were anesthetized by an intraperitoneal injection of Zoletil (30 mg/kg; Virvac Laboratories, Carros, France) and 200 μL of 5% Evans blue was administered into the jugular vein. After 10 min, the mice were sacrificed, and the brains were isolated immediately. For quantitative measurement, brains were homogenized in sample tubes, with each containing 500 μL of formamide (Sigma-Aldrich). Samples were incubated at 55 °C for 24 h to extract the Evans blue from the brain tissue. The mixture was centrifuged, and absorbance of the supernatants was measured at 610 nm using a VERSASA max microplate reader (Molecular Devices). The quantitative graph is expressed as the amount of Evans blue (ng) per tissue amount (mg).

### Western blot analysis

Mice were anesthetized with Zoletil (30 mg/kg) and transcardially perfused with 0.9% normal saline. The hippocampus was isolated for western blot analyses. Tissues in radioimmunoprecipitation assay buffer (BIOSESANG, INC., Seongnam, South Korea) with with Halt^TM^ protease and Phosphatase inhibitor cocktail (1:100; Thermo Fisher Scientific) were homogenized with homogenizing pestles on ice. After the homogenates were centrifuged at 13,000 rpm at 4 °C for 20 min, the protein concentrations in each supernatant were determined by using a Pierce^®^ BCA protein assay kit (Thermo Fisher Scientific). Proteins were denatured at 95 °C for 5 min, electrophoresed using 6-10% SDS polyacrylamide gel electrophoresis, and transferred to polyvinylidene difluoride membranes (Merck Millipore, Bedford, MA, USA). The blots were blocked with 5% bovine serum albumin (BSA) for 1 h at room temperature and incubated with rabbit anti-Iba-1 (1:500; Santa Cruz Biotechnology, Santa Cruz, CA, USA), rabbit anti-CD11b (1:1000; Merck Millipore), and rabbit anti-NE (1:1000; Abcam, Cambridge, UK) overnight at 4 °C. The horseradish peroxidase (HRP)-conjugated anti-rabbit, anti-goat, or anti-mouse immunoglobulin (Ig) G reagents, diluted in 2% BSA, were used as secondary antibodies (Jackson ImmunoRearch Laboratories, West Grove, PA, USA). The bands were detected with an enhanced chemiluminescence system (West-Q pico Dura ECL solutions; GenDEPOT, Barker, TX, USA) using the LAS 4000 program (GE Healthcare, Pittsburgh, PA, USA). The relative optical density of the bands was scanned and quantified by densitometry using the Multi Gauge V3.0 software (Fuji photo film Co., Ltd., Japan).

### Immunofluorescence staining

Mouse brains were fixed with 4% formaldehyde and cut into 20-μm coronal sections with a cryotome (Thermo Fisher scientific). After washing with PBS, sections were permeabilized with Triton-X 100 (0.3% diluted in PBS) for 1 h at room temperature. The tissue sections were placed onto coated slides and blocked with 5% BSA for 1 h at room temperature. Then, the sections were treated respectively with the following primary antibodies overnight at 4 °C: rabbit-E-selectin (H-300) (1:60; Santa Cruz Biotechnology), goat anti-ICAM-1 (M-19) (1:60; Santa Cruz Biotechnology), mouse anti-P-selectin (1:60; Santa Cruz Biotechnology), goat anti-VCAM-1 (C-19) (1:60; Santa Cruz Biotechnology), rabbit anti- NE (1:100; Abcam), goat anti-MPO (1:100; R&D systems, MN, USA), and goat anti-Iba-1 (1:100; Abcam). Fluorescdin isothiocyanate (FITC) or rhodamine-conjugated secondary antibody was used (1:1000, Jackson ImmunoResearch). Tissues were mounted with Vectashield with 4′,6-diamidino-2-phenylindole (DAPI; Vector Laboratories, Inc., Burlingame, CA, USA). Images were acquired using an LSM 710 confocal laser scanning microscope (Carl Zeiss) at excitation/emission wavelengths of 355/463 nm for DAPI, 495/517 nm for FITC, and 551/573 nm for rhodamine by the smart setup system in the Zen microscope and image software (Carl Zeiss, Thornwood, NY, USA). Three or four acquisition areas were selected randomly. The mean intensities for E-selectin, ICAM-1, p-selectin, and VCAM-1 immunoreactivity were analyzed using Zen 2010 software (Carl Zeiss). Semi-quantitative analyses were performed using the confocal images and Zen 2010 software.

### Cresyl violet staining

After cardiac reperfusion, the mouse brains were isolated and fixed with 3.7% formaldehyde. Cresyl violet acetate (0.5%) (Sigma-Aldrich) was dissolved in 300 mL of distilled water with 10% glacial acetic acid and filtered with filter paper. Brains were stained in cresyl violet solution for three times. Slides were coverslipped with permanent mounting medium (Vector Laboratories, Inc., Burlingame, CA, USA), air-dried, and observed under a microscope (Olympus, Tokyo, Japan).

### Microglia/macrophage cell cultures

Mouse brain microglial cells (BV-2) were cultured with RPMI 1640 (Hyclone™, GE Healthcare Life Sciences, Logan, UT, USA) containing 10% fetal bovine serum (GE Healthcare Life Sciences) and 1% penicillin-streptomycin solution (Thermo Fisher Scientific). A mouse macrophage cell line (RAW 264.7) was purchased from the Korean Cell Line Bank (KCIB, Seoul, Korea) and cultured with Dulbecco’s Modified Eagle Medium high glucose cultured media (Hyclone™, GE Healthcare Life Sciences), 10% fetal bovine serum (GE Healthcare Life Sciences), and 1% penicillin-streptomycin solution (Thermo Fisher Scientific). The cultured cells were incubated in a humid atmosphere under 5% CO_2_ at 37 °C.

### LPS and nt-p65-TMD treatment

Microglial and macrophage cells were seeded in six-well plates at a density of 1.6 × 10^6^ at 24 h before the experiments. We delivered 500 nM or 1 μM of the fusion protein nt-p65-TMD to the cells for 2 h prior to LPS treatment (1 μg/mL). The LPS was then added for 1 h and the culture medium was replaced. After a 24-h incubation period, the microglia/macrophages and cell media were collected and stored at −70 °C until ready for analysis.

### Enzyme-linked immunosorbent assays for interleukin-1 beta, interleukin-6, and tumor necrosis factor-alpha

The levels of pro-inflammatory cytokines such as IL-1β, IL-6, and TNF-α were detected in the hippocampus and spleen from the mice and in the microglia/macrophage cell-conditioned medium. Samples were analyzed with a high-sensitivity mouse IL-1β/IL-1F2, IL-6, TNF-α Quantikine enzyme-linked immunosorbent assay (ELISA) kit (R&D systems). According to the manufacturer’s instructions, the hippocampus or spleen samples, reagents, and standards were prepared, and the standard solution, control, and samples with Assay Diluents were added to each well and incubated. After washing with wash buffer, mouse IL-1β, IL-6, or TNF-α conjugates were added, followed by incubation for 2 h at room temperature. The levels were estimated in each well using an automatic ELISA reader at wavelength of 450 nm.

### Quantitative real time-PCR

The hippocampus and spleen from mice and the microglial/macrophage cells in each group were rapidly dissected or collected and stored at −70 °C until real-time PCR. Total RNA was extracted using HiGene™ Total RNA Prep kit (BIOFACT, Daejeon, Korea) following the manufacturer’s instructions. Tissues (<30 mg) were lysed in RB solution with β-mercaptoethanol and proteinase K. After the samples were centrifuged at 14,000 rpm for 3 min at 4 °C, ethanol (100%) was added, and the mixture was vortexed for 30 s. The RNA was eluted with 50–100 μL of RNase-free water. One-step real-time PCR was performed using the One-step SYBR PrimeScript RT-PCR Kit II (Perfect Real Time; Takara Bio Inc, JAPAN) on an ABI StepOnePlus Real-Time PCR system (Applied Biosystems, Thermo Fisher Scientific). The concentration of RNA was measured at 260/280 nm using NanoDrop® ND-1000 (Thermo Fisher Scientific). The primers were purchased from Bioneer (Daejeon, Korea) (Supplementary Table 1). PCR was performed in a total reaction mixture volume of 20 μL, which was composed of One-step SYBR RT-PCR Buffer, PrimeScript 1 step Enzyme Mix 2, ROX Reference Dye, each forward primer, or each reverse primer, and sample RNA diluted in RNase Free distilled water according to manufacturer’s protocol. The PCR cycling system was set as follows: reverse transcription was performed for 5 min at 42 °C and for 10 s at 95 °C; the PCR reaction was performed for 40 cycles of 5 s at 95 °C and for 34 s at 60 °C; and the final step was performed at 95 °C for 15 s, 60 °C for 1 min, and 95 °C for 15 s. Cycling threshold values were normalized to the cycling threshold values of β-actin. The results were analyzed with the StepOneSoftware v2.3 (Thermo Fisher Scientific).

### Immunocytochemistry

The microglia and macrophages were seeded onto coated slides and fixed with 4% formaldehyde. Cells were washed three times with PBS, and then blocked with 5% BSA for 1 h at room temperature. The transduced nt-p65-TMD was stained with anti-FLAG-FITC (1:100, Sigma-Aldrich) overnight at 4 °C. After washing with PBS, the slides were mounted with Vectashield with DAPI (Vector Laboratories, Inc.) and observed under an LSM 710 confocal microscope (Carl Zeiss) (Supplementary Fig. S1d).

### Isolation of microglia/macrophages and flow cytometric analysis

The fresh hippocampus and spleen were placed in a Petri dish on ice and mechanically chopped into small pieces with Dulbecco’s PBS using razor blades. FACS buffer (Dulbecco’s PBS with 2% fetal bovine serum and, 0.09% sodium azide; BD Pharmigen^TM^, BD Biosciences, San Jose, CA, USA) containing 0.5 mg/mL of type I collagenase was added to the tissue pieces and incubated for 10 min. After a dissociation process, the cells were centrifuged at 250 × g for 3 min, and the pellet was resuspended with 500 μL FACS buffer. After collecting the pellets, mononuclear cells were blocked with mouse Fc blocking solution (1:50; BD Pharmigen^TM^, BD Biosciences) for 10 min on ice. Next, cells were centrifuged at 250 × g for 3 min at 4 °C and the supernatant was discarded. Cells were respectively labeled with the following antibodies for 30 min on ice: mouse anti-CD11b-APC (1:50; BD Pharmigen^TM^, BD Biosciences), mouse anti-CD45-PerCP-Cy^TM^ 5.5 (1:50; BD Pharmigen^TM^, BD Biosciences), mouse anti-CD86-FITC (1:50; MACS Miltenyi Biotec, Bergisch Gladbach, Germany), and mouse anti-CD206-PE (1:50; BD Pharmigen^TM^, BD Biosciences). Then, the cell suspensions were filtered through a cell strainer with a 40-µm nylon mesh. Cell fluorescence was acquired by flow cytometry with an LSR II analyzer (BD Pharmigen^TM^, BD Biosciences). The isolated cells were then stained with the pro-inflammatory marker CD86 and the anti-inflammatory marker CD206, respectively. We defined pro-inflammatory microglia/macrophages as CD86^+^ and anti-inflammatory microglia/macrophages as CD206^+^ among CD11b^+^/CD45^+^ cells. Data were analyzed using FlowJo version10 software (FLOWJO, LLC, Ashland, OR, USA).

### Statistical analysis

Data are presented as the means ± standard error of the mean (SEM). Behavioral data are expressed as medians and interquartile ranges. Statistical comparisons among the groups were assessed with a one-way analysis of variance (ANOVA) followed by Bonferroni tests. Statistical comparisons in passive avoidance test were calculated by non-parametric Kruskal-Wallis with Dunn’s multiple comparisons. (Prism version 5.0 software, GraphPad Software, SanDiego, CA, USA). Statistical significance was set at p-values less than 0.05.

## Electronic supplementary material

Supplementary information
