# Supplementary material for: Cell-penetrating interactomic inhibition of nuclear factor-kappa B in a mouse model of postoperative cognitive dysfunction
Source: Sci Rep. 2017 Oct 18;7:13482. doi: 10.1038/s41598-017-14027-2 (PMC5647420; doi:10.1038/s41598-017-14027-2)
Supplement: Supplementary file 1 — Supplementary information [file 41598_2017_14027_MOESM1_ESM.pdf]

[Supplementary Information]

Cell-penetrating interactomic inhibition of nuclear factor-kappa B in a mouse model of postoperative cognitive dysfunction

So Yeong Cheon[1,2†], Jeong Min Kim[1,2†], Eun Hee Kam[1,2], Chun-Chang Ho[3], Eun Jung Kim[1,2], Seungsoo Chung[4], Ji-Hyun Jeong[4], Diane Da-Hyun Lee[5,6], Sang-Won Lee[5], and Bon-Nyeo Koo[1,2*]

[1]Department of Anesthesiology and Pain Medicine, Yonsei University College of Medicine, Seoul, Republic of Korea

[2]Anesthesia and Pain Research Institute, Yonsei University College of Medicine, Seoul, Republic of Korea

[3]Department of Biotechnology, College of Life Science and Biotechnology, Yonsei University, Seoul, Republic of Korea

[4]Department of Physiology, Brain Korea 21 Plus Project for Medical Science, Yonsei University College of Medicine, Seoul, Republic of Korea

[5]Division of Rheumatology, Department of Internal Medicine, Yonsei University College of Medicine, Seoul, Republic of Korea

[6]Seoul International School, Seongnam, Republic of Korea

***Correspondence should be addressed to:**

Bon-Nyeo Koo, MD, PhD. Department of Anesthesiology and Pain Medicine, Yonsei University College of Medicine, 50-1, Yonsei-ro, Seodaemun-gu, Seoul 120-752, Republic of Korea, E-mail: koobn@yuhs.ac

[Supplementary data]

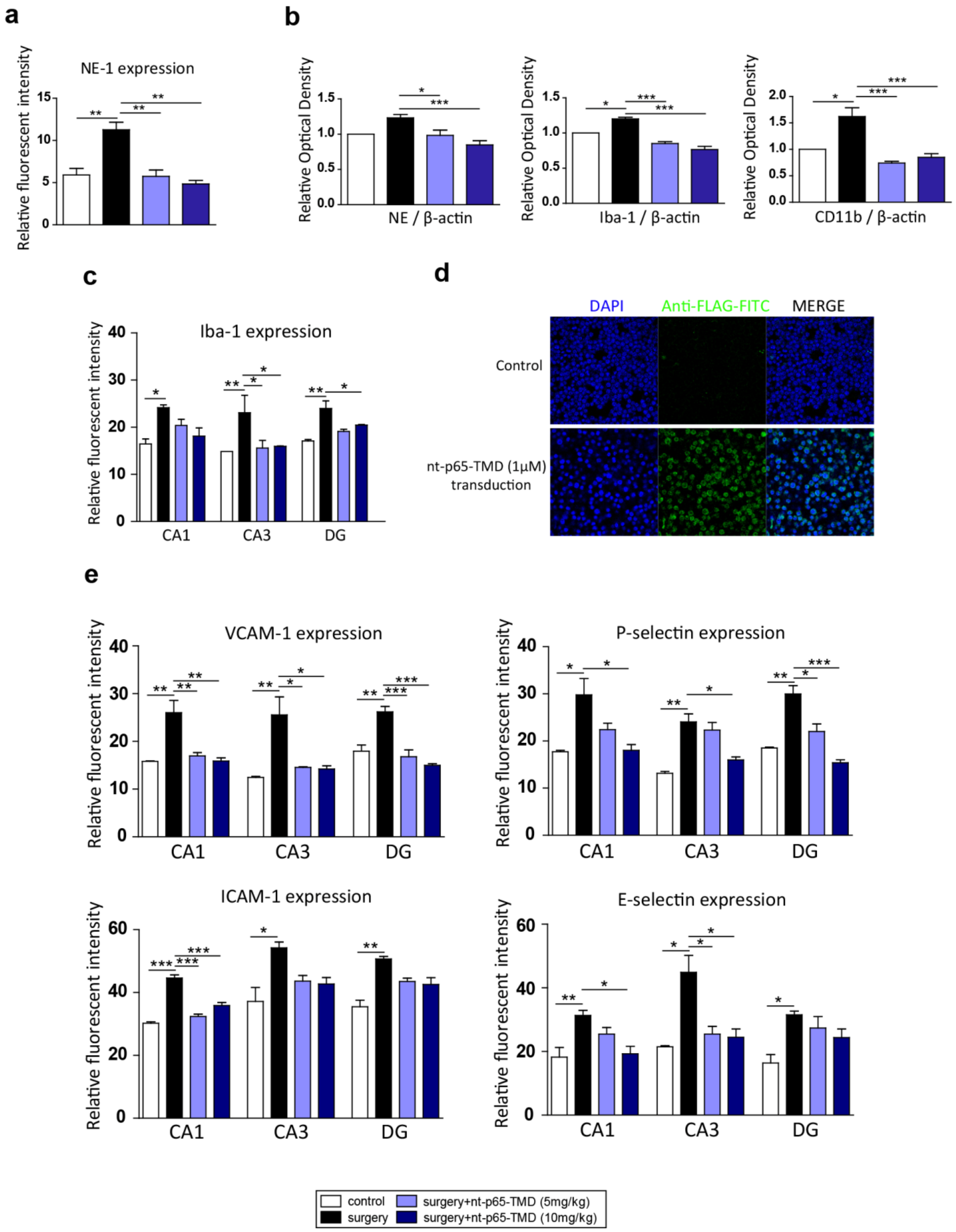

Supplementary Figure S1. (a) The graph shows that relative fluorescent intensity for NE-1 in the cerebral cortex at 2 days after surgery (n = 3). (b) Protein levels of NE (n = 5-11), Iba-1 (n = 4-11), and CD11b (n = 4-11) in the Hippocampus was measured by western blot analysis

at 2 days after surgery and semi-quantative graphs shows relative optical desity. Each value was normalized with β-actin. (c) Expression of Iba-1 in the hippocampus was calculated by immunoreactivity with Carl zeiss Zen program 2010 (n = 3-4). (d) Cell transduction of nt-p65-TMD was confirmed by immunocytochemistriy for FLAG in RAW264.7 cell line. (e) VCAM-1 (n = 3-4), ICAM-1 (n = 3-4), P-selectin (n = 3-5), and E-selectin (n = 3-5) expressions in the hippocampus were measured by immunofluorescent staining at 2 days after surgery. Values are means ± SEM. *P* values were calculated with multiple comparisons by Bonferroni tests. *$p < 0.05$, **$p < 0.01$, ***$p < 0.001$ vs. surgery group. Statistical parameter (Supplementary Table 8).

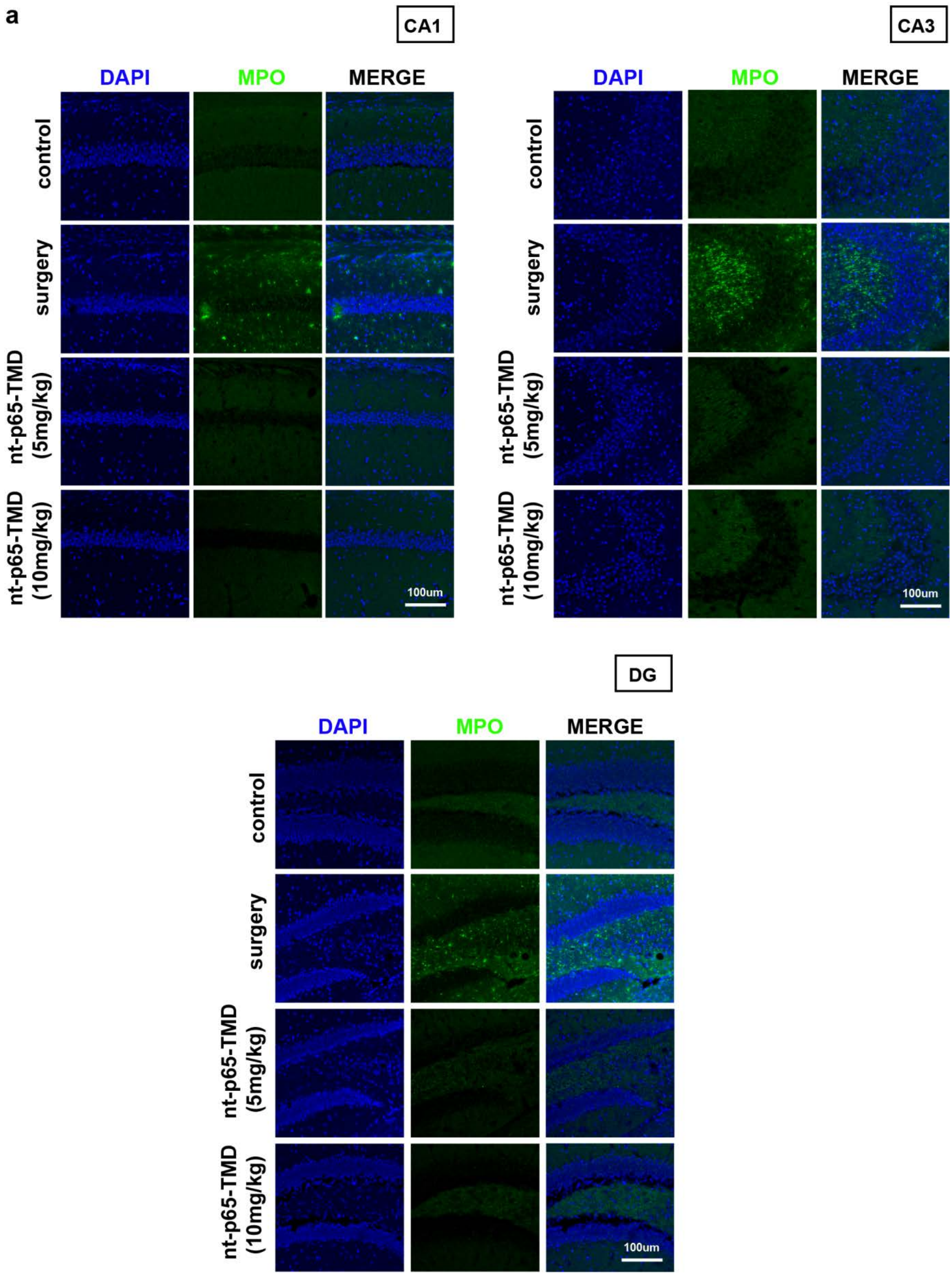

Supplementary Figure S2. (a) Immunofluorescent images show MPO expression in the hippocampus.

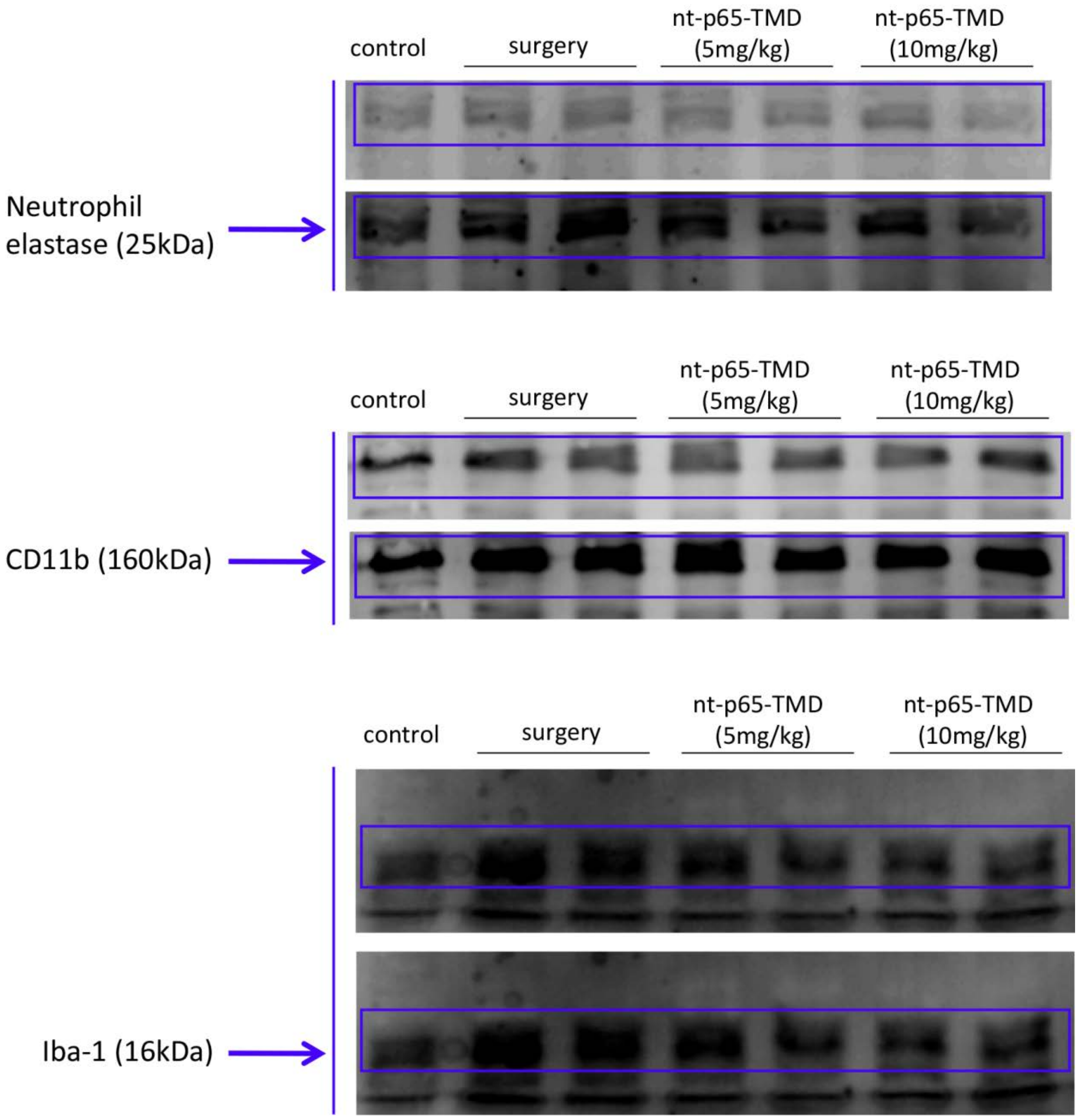

Supplementary Figure S3. Full size-blots of Fig. 2e

Table. 1. Sequence of primer for real-time PCR

β-actin	Forward	5' - AGAGGGAAATCGTGCGTGAC - 3'
	Reverse	5' - CAATAGTGATGACCTGGCCGT -3'
TNF-α	Forward	5' - ACGGCATGGATCTCAAAGAC - 3'
	Reverse	5' - AGATAGCAAATCGGCTGACG - 3'
IL-1β	Forward	5' - TGTCTTGGCCGAGGACTAAG - 3'
	Reverse	5' - TGGGCTGGACTGTTTCTAATGC -3'
IL-6	Forward	5' - TCCAGTTGCCTTCTTGGGAC - 3'
	Reverse	5' - GTGTAATTAAGCCTCCGACTTG - 3'
iNOS	Forward	5' - CAGCTGGGCTGTACAAACCT - 3'
	Reverse	5' - CATTGGAAGTGAAGCGTTTCG - 3'
IL-10	Forward	5' - GCTCTTACTGACTGGCATGAG - 3'
	Reverse	5' - CGCAGCTCTAGGAGCATGTG - 3'
Arginase-1	Forward	5' - GAACACGGCAGTGGCTTTAAC - 3'
	Reverse	5' - TGCTTAGCTCTGTCTGCTTTGC - 3'
IL-17A	Forward	5' - AACACTGAGGCCAAGGACTT - 3'
	Reverse	5' - ACCCACCAGCATCTTCTCG - 3'
I-CAM	Forward	5' - GGCTGGCATTGTTCTCTAA - 3'
	Reverse	5' - TTCAGAGGCAGGAAACAGG - 3'
V-CAM	Forward	5' - CCCAAACAGAGGCAGAGTGT - 3'
	Reverse	5' - CAGGATTTTGGGAGCTGGTA - 3'
E-selectin	Forward	5' - AGCTACCCATGGAACACGAC - 3'
	Reverse	5' - CGCAAGTTCTCCAGCTGTT - 3'
P-selectin	Forward	5' - ATGCCTGGCTACTGGACACT - 3'
	Reverse	5' - CTTCATCGCACATGAACTGG - 3'
GAPDH	Forward	5' - CCATTTGCAGTGGCAAAG - 3'
	Reverse	5' - CACCCCATTTGATGTTAGTG - 3'

Supplementary Table 1. Sequence of primers for real-time PCR.

Table 2. Statistical parameter in Figure 1.

Passive avoidance test

Before surgery	control	surgery	surgery+nt-p65-TMD (5mg/kg)	surgery+nt-p65-TMD (10mg/kg)
Minimum	6	4	5	4
25% Percentile	7	8	7	6
Median	10.5	11	10	11
75% Percentile	15.75	21	11	20
Maximum	38	23	21	27
**After surgery**	**control**	**surgery**	**surgery+nt-p65-TMD (5mg/kg)**	**surgery+nt-p65-TMD (10mg/kg)**
Minimum	74	13	12	15
25% Percentile	127.5	28	29	92
Median	262	78	128	257
75% Percentile	300	123	204	300
Maximum	300	251	300	300

Elevated Plus Maze

Before surgery	control	surgery	surgery+nt-p65-TMD (5mg/kg)	surgery+nt-p65-TMD (10mg/kg)
Minimum	15.45	10.95	9.72	15.42
25% Percentile	19.5	16.25	15.45	19.61
Median	24.32	20.48	22.35	27.04
75% Percentile	31.57	26.9	25.87	41.07
Maximum	41.05	41.31	46.13	50.38
**After surgery**	**control**	**surgery**	**surgery+nt-p65-TMD (5mg/kg)**	**surgery+nt-p65-TMD (10mg/kg)**
Minimum	2.55	7.68	4.38	3.42
25% Percentile	4.05	14.3	5.765	5.575
Median	7.78	17.72	10.92	8.35
75% Percentile	9.085	32.22	14.13	12.24
Maximum	17.98	46.26	16.85	18.22
**Learning index**	**control**	**surgery**	**surgery+nt-p65-TMD (5mg/kg)**	**surgery+nt-p65-TMD (10mg/kg)**
Minimum	9	-15.64	-1.2	7.87
25% Percentile	11.29	-10.14	7.3	13.62
Median	14.8	-0.17	12.27	18.75
75% Percentile	27.52	9.385	16.62	26.19
Maximum	38.5	10.54	32.06	46.96

Novel objective recognition test

	control	surgery	surgery+nt-p65-TMD (5mg/kg)	surgery+nt-p65-TMD (10mg/kg)
Minimum	54.62	36.97	40.71	52.74
25% Percentile	61.5	38.51	47.6	59.69
Median	72.5	49.87	53.8	67.81
75% Percentile	78.07	55.24	68.27	72.8
Maximum	81.51	58.9	71.06	77.52

Electrophysiology

	control	surgery	surgery+nt-p65-TMD (5mg/kg)	surgery+nt-p65-TMD (10mg/kg)
Mean	147.4	106.8	132.3	141.7
Std. Error	5.56	4.119	4.049	3.743

Supplementary Table 2. Statistical parameter in Figure 1.

Table 3. Statistical parameter in Figure 2.

Evans Blue Extravasation

striatum & cortex	control	surgery	surgery+nt-p65-TMD (5mg/kg)	surgery+nt-p65-TMD (10mg/kg)
Mean	6.326	16.37	10.26	9.089
Std. Error	1.484	3.135	0.739	1.515
**Hippocampus**	**control**	**surgery**	**surgery+nt-p65-TMD (5mg/kg)**	**surgery+nt-p65-TMD (10mg/kg)**
Mean	9.245	16.52	13.25	8.029
Std. Error	0.5631	0.7432	0.3868	1.515

MPO expression (IF)

CA1	control	surgery	surgery+nt-p65-TMD (5mg/kg)	surgery+nt-p65-TMD (10mg/kg)
Mean	12.65	33.79	15.91	14.92
Std. Error	0.3499	3.247	0.5455	1.575
**CA3**	**control**	**surgery**	**surgery+nt-p65-TMD (5mg/kg)**	**surgery+nt-p65-TMD (10mg/kg)**
Mean	14.05	35.7	16.31	15.64
Std. Error	1.002	3.052	0.3507	2.377
**DG**	**control**	**surgery**	**surgery+nt-p65-TMD (5mg/kg)**	**surgery+nt-p65-TMD (10mg/kg)**
Mean	16.42	45.89	11.94	16.02
Std. Error	1.417	1.574	0.9182	1.318

Real-time PCR

ICAM-1	control	surgery	surgery+nt-p65-TMD (5mg/kg)	surgery+nt-p65-TMD (10mg/kg)
Mean	0.937	4.725	1.162	1.349
Std. Error	0.03901	2.102	0.1675	0.09472
**VCAM-1**	**control**	**surgery**	**surgery+nt-p65-TMD (5mg/kg)**	**surgery+nt-p65-TMD (10mg/kg)**
Mean	0.8401	1.402	1.075	0.9462
Std. Error	0.07853	0.1575	0.08977	0.0839
**E-selectin**	**control**	**surgery**	**surgery+nt-p65-TMD (5mg/kg)**	**surgery+nt-p65-TMD (10mg/kg)**
Mean	0.9737	1.774	1.258	1.079
Std. Error	0.07927	0.1958	0.1907	0.1915
**P-selectin**	**control**	**surgery**	**surgery+nt-p65-TMD (5mg/kg)**	**surgery+nt-p65-TMD (10mg/kg)**
Mean	0.8337	8.417	0.8303	1.4
Std. Error	0.03283	4.36	0.07995	0.277

Supplementary Table 3. Statistical parameter in Figure 2.

Table 4. Statistical parameter in Figure 3.

ELISA assay (Hippocampus)

IL-1β	control	surgery	surgery+nt-p65-TMD (5mg/kg)	surgery+nt-p65-TMD (10mg/kg)
Mean	45660000	68640000	35320000	39270000
Std. Error	318756	5122000	2819000	1725000
**IL-6**	control	surgery	surgery+nt-p65-TMD (5mg/kg)	surgery+nt-p65-TMD (10mg/kg)
Mean	6922000	37120000	16650000	12630000
Std. Error	367577	11870000	2183000	1165000
**TNF-α**	control	surgery	surgery+nt-p65-TMD (5mg/kg)	surgery+nt-p65-TMD (10mg/kg)
Mean	30630000	52710000	32890000	38020000
Std. Error	3252000	3208000	4909000	3779000
**MCP-1**	control	surgery	surgery+nt-p65-TMD (5mg/kg)	surgery+nt-p65-TMD (10mg/kg)
Mean	27130000	39740000	28070000	30610000
Std. Error	3976000	1083000	2132000	1699000

ELISA assay (Spleen)

IL-1β	control	surgery	surgery+nt-p65-TMD (5mg/kg)	surgery+nt-p65-TMD (10mg/kg)
Mean	77180	122641	63524	65625
Std. Error	4408	13618	2822	5785
**IL-6**	control	surgery	surgery+nt-p65-TMD (5mg/kg)	surgery+nt-p65-TMD (10mg/kg)
Mean	2642000	3988000	1653000	2059000
Std. Error	245834	278022	450682	454569
**TNF-α**	control	surgery	surgery+nt-p65-TMD (5mg/kg)	surgery+nt-p65-TMD (10mg/kg)
Mean	5117000	26320000	8349000	5873000
Std. Error	1235000	6588000	2133000	1003000

Spleen weight (mg)

	control	surgery	surgery+nt-p65-TMD (5mg/kg)	surgery+nt-p65-TMD (10mg/kg)
Mean	139.3	96.92	113.6	133.1
Std. Error	8.016	8.577	8.972	14.34

Spleen size (mm)

	control	surgery	surgery+nt-p65-TMD (5mg/kg)	surgery+nt-p65-TMD (10mg/kg)
Mean	21.2	17.85	18.36	19.62
Std. Error	0.2795	0.4058	0.6364	0.8737

Supplementary Table 4. Statistical parameter in Figure 3

Table 5. Statistical parameter in Figure 4.

Real-time PCR (Hippocampus)

IL-1β	control	surgery	surgery+nt-p65-TMD (10mg/kg)
Mean	0.6908	1.183	0.6126
Std. Error	0.07517	0.1521	0.09014
**IL-6**	**control**	**surgery**	**surgery+nt-p65-TMD (10mg/kg)**
Mean	0.7525	1.307	0.6104
Std. Error	0.09554	0.1614	0.0244
**TNF-α**	**control**	**surgery**	**surgery+nt-p65-TMD (10mg/kg)**
Mean	0.6331	2.605	0.6194
Std. Error	0.1223	1.115	0.1132
**arginase-1**	**control**	**surgery**	**surgery+nt-p65-TMD (10mg/kg)**
Mean	7.647	8.223	8.037
Std. Error	0.1719	0.2414	0.4386
**IL-10**	**control**	**surgery**	**surgery+nt-p65-TMD (10mg/kg)**
Mean	8.379	8.955	8.515
Std. Error	0.2117	0.2894	0.4621

Real-time PCR (Spleen)

IL-1β	control	surgery	surgery+nt-p65-TMD (10mg/kg)
Mean	0.7312	3.823	0.9957
Std. Error	0.1133	0.5327	0.103
**IL-6**	**control**	**surgery**	**surgery+nt-p65-TMD (10mg/kg)**
Mean	0.7372	1.277	0.8521
Std. Error	0.03471	0.04705	0.05917
**TNF-α**	**control**	**surgery**	**surgery+nt-p65-TMD (10mg/kg)**
Mean	0.5838	2.036	0.461
Std. Error	0.09839	0.3099	0.1137
**arginase-1**	**control**	**surgery**	**surgery+nt-p65-TMD (10mg/kg)**
Mean	1.128	0.9996	1.186
Std. Error	0.3234	0.1377	0.4583
**IL-10**	**control**	**surgery**	**surgery+nt-p65-TMD (10mg/kg)**
Mean	2.059	0.5546	2.122
Std. Error	0.5482	0.157	0.4774

Supplementary Table 5. Statistical parameter in Figure 4.

Table 6. Statistical parameter in Figure 5.

Passive avoidance test

Before LPS	control	LPS	LPS+nt-p65-TMD (5mg/kg)
Minimum	3	7	8
25% Percentile	5.25	7.5	9.5
Median	8.5	9	17
75% Percentile	12.75	20.5	21
Maximum	15	25	23
After LPS	control	LPS	LPS+nt-p65-TMD (5mg/kg)
Minimum	125	16	67
25% Percentile	194.5	47	81
Median	281.5	95	182
75% Percentile	300	210.5	300
Maximum	300	300	300

Elevated Plus Maze

Before LPS	control	LPS	LPS+nt-p65-TMD (5mg/kg)
Minimum	18.88	6.32	17.55
25% Percentile	24.86	12.18	18.95
Median	28.5	18.78	33.25
75% Percentile	33.93	25.78	47.32
Maximum	41.05	33.38	55.18
After LPS	control	LPS	LPS+nt-p65-TMD (5mg/kg)
Minimum	2.55	12.75	2.18
25% Percentile	4.328	13.08	4.08
Median	5.77	29.98	9.45
75% Percentile	7.858	52.02	12.35
Maximum	9.68	177.2	23.55
Learning index	control	LPS	LPS+nt-p65-TMD (5mg/kg)
Minimum	9.2	-156.4	7.7
25% Percentile	18.32	-43.76	10.7
Median	21.85	-1.77	23.8
75% Percentile	28.4	3.37	31.63
Maximum	28.4	3.4	45.14

Novel objective recognition test

	control	LPS	LPS+nt-p65-TMD (5mg/kg)
Minimum	42.22	20.41	35.32
25% Percentile	47.69	25.63	36.54
Median	53.23	37.89	51.62
75% Percentile	66.25	48.7	59.06
Maximum	73.53	49.93	68.88

Electrophysiology

	control	LPS	LPS+nt-p65-TMD (5mg/kg)
Mean	49.47	11.24	39.68
Std. Error	3.315	2.691	2.039

Table 6. Statistical parameter in Figure 5.

ELISA assay (Hippocampus)

IL-1β	control	LPS	LPS+nt-p65-TMD (5mg/kg)
Mean	11.5	63.93	37.8
Std. Error	5.796	2.937	4.885
IL-6	control	LPS	LPS+nt-p65-TMD (5mg/kg)
Mean	7.611	18.64	8.469
Std. Error	1.855	1.673	2.057

ELISA assay (Spleen)

IL-1β	control	LPS	LPS+nt-p65-TMD (5mg/kg)
Mean	0.4045	30.06	27.65
Std. Error	0.1041	1.762	2.243
IL-6	control	LPS	LPS+nt-p65-TMD (5mg/kg)
Mean	29.12	125.2	61.95
Std. Error	0.9818	35.49	11.84

Real-time PCR (Hippocampus)

IL-1β	control	LPS	LPS+nt-p65-TMD (5mg/kg)
Mean	1.097	16.26	1.132
Std. Error	0.122	6.81	0.1322
IL-6	control	LPS	LPS+nt-p65-TMD (5mg/kg)
Mean	0.9371	5.599	0.5839
Std. Error	0.04727	2.231	0.04991
TNF-α	control	LPS	LPS+nt-p65-TMD (5mg/kg)
Mean	0.8365	86.32	5.345
Std. Error	0.09438	45.28	0.8527
IL-10	control	LPS	LPS+nt-p65-TMD (5mg/kg)
Mean	1.16	1.437	1.324
Std. Error	0.112	0.3374	0.1389

Real-time PCR (Spleen)

IL-1β	control	LPS	LPS+nt-p65-TMD (5mg/kg)
Mean	0.5141	1.708	0.1277
Std. Error	0.05109	0.4854	0.02167
IL-6	control	LPS	LPS+nt-p65-TMD (5mg/kg)
Mean	0.6111	1.218	0.1128
Std. Error	0.1229	0.2136	0.01992
TNF-α	control	LPS	LPS+nt-p65-TMD (5mg/kg)
Mean	0.5072	2.883	0.2632
Std. Error	0.119	1.185	0.06191
IL-10	control	LPS	LPS+nt-p65-TMD (5mg/kg)
Mean	1.339	0.8091	3.307
Std. Error	0.3379	0.05862	1.001

Supplementary Table 6. Statistical parameter in Figure 5

Table 7. Statistical parameter in Figure 6.

ELISA assay (BV2 cell line)

IL-1β	control	LPS	LPS+nt-p65-TMD (500nM)	LPS+nt-p65-TMD (1μM)
Mean	0.5294	21.21	14.64	11.25
Std. Error	0.4647	1.375	3.239	1.066
IL-6	control	LPS	LPS+nt-p65-TMD (500nM)	LPS+nt-p65-TMD (1μM)
Mean	576.7	11064	10961	9752
Std. Error	26.09	362.9	235.6	283.4
TNF-α	control	LPS	LPS+nt-p65-TMD (500nM)	LPS+nt-p65-TMD (1μM)
Mean	247.5	960.2	792.2	671.9
Std. Error	8.566	19.57	18.59	33.65

Real-time PCR (BV2 cell line)

IL-1β	control	LPS	LPS+nt-p65-TMD (500nM)	LPS+nt-p65-TMD (1μM)
Mean	0.8055	2.151	1.426	0.2537
Std. Error	0.06905	0.2131	0.1847	0.07577
IL-6	control	LPS	LPS+nt-p65-TMD (500nM)	LPS+nt-p65-TMD (1μM)
Mean	0.97	5.381	4.449	3.492
Std. Error	0.1312	0.4023	1.109	0.9553
TNF-α	control	LPS	LPS+nt-p65-TMD (500nM)	LPS+nt-p65-TMD (1μM)
Mean	1.183	3.075	2.35	2.164
Std. Error	0.1719	0.08774	0.2702	0.2601
CXCL10	control	LPS	LPS+nt-p65-TMD (500nM)	LPS+nt-p65-TMD (1μM)
Mean	0.9371	4.958	3.473	2.809
Std. Error	0.1264	0.4874	0.5617	0.5822
iNOS	control	LPS	LPS+nt-p65-TMD (500nM)	LPS+nt-p65-TMD (1μM)
Mean	1.014	2.689	1.948	1.348
Std. Error	0.03082	0.1258	0.1136	0.244
arginase-1	control	LPS	LPS+nt-p65-TMD (500nM)	LPS+nt-p65-TMD (1μM)
Mean	1.081	0.7841	0.8235	0.8576
Std. Error	0.1118	0.142	0.06377	0.1177
IL-10	control	LPS	LPS+nt-p65-TMD (500nM)	LPS+nt-p65-TMD (1μM)
Mean	1.053	0.7651	0.8733	0.8004
Std. Error	0.09778	0.06243	0.06142	0.06796

.

Table 7. Statistical parameter in Figure 6.

ELISA assay (RAW264.7 cell line)

IL-1β	control	LPS	LPS+nt-p65-TMD (500nM)	LPS+nt-p65-TMD (1μM)
Mean	4.027	651.8	513.3	451.5
Std. Error	1.26	19.05	29.09	31.87
**IL-6**	**control**	**LPS**	**LPS+nt-p65-TMD (500nM)**	**LPS+nt-p65-TMD (1μM)**
Mean	-38.69	15064	12728	12926
Std. Error	3.248	779.1	156.6	128.1
**TNF-α**	**control**	**LPS**	**LPS+nt-p65-TMD (500nM)**	**LPS+nt-p65-TMD (1μM)**
Mean	79.42	1486	1405	1386
Std. Error	2.786	26.53	8.123	6.732

Real-time PCR (RAW264.7 cell line)

IL-1β	control	LPS	LPS+nt-p65-TMD (500nM)	LPS+nt-p65-TMD (1μM)
Mean	1.82	390.2	276.2	76.42
Std. Error	0.2967	51.22	51.29	21.14
**IL-6**	**control**	**LPS**	**LPS+nt-p65-TMD (500nM)**	**LPS+nt-p65-TMD (1μM)**
Mean	5.839	468.1	293.1	246.1
Std. Error	1.977	92.41	23.38	38.93
**TNF-α**	**control**	**LPS**	**LPS+nt-p65-TMD (500nM)**	**LPS+nt-p65-TMD (1μM)**
Mean	1.069	9.166	8.922	3.847
Std. Error	0.03002	0.2636	0.7214	0.2385
**CXCL10**	**control**	**LPS**	**LPS+nt-p65-TMD (500nM)**	**LPS+nt-p65-TMD (1μM)**
Mean	1.129	6.429	3.065	2.576
Std. Error	0.07734	0.612	0.2363	0.3062
**iNOS**	**control**	**LPS**	**LPS+nt-p65-TMD (500nM)**	**LPS+nt-p65-TMD (1μM)**
Mean	1.49	3563	3521	1732
Std. Error	0.1949	704.9	859.7	466.1
**arginase-1**	**control**	**LPS**	**LPS+nt-p65-TMD (500nM)**	**LPS+nt-p65-TMD (1μM)**
Mean	2.06	3.769	5.093	10.05
Std. Error	1.003	1.475	3.067	3.821
**IL-10**	**control**	**LPS**	**LPS+nt-p65-TMD (500nM)**	**LPS+nt-p65-TMD (1μM)**
Mean	4.212	2.595	7.324	14.01
Std. Error	2.036	0.9585	2.907	3.352

Supplementary Table 7. Statistical parameter in Figure 6

Table 8. Statistical parameter in Supplementary Figure 1.

NE expression (IF)

cortex	control	surgery	surgery+nt-p65-TMD (5mg/kg)	surgery+nt-p65-TMD (10mg/kg)
Mean	5.907	11.27	5.739	4.841
Std. Error	0.7683	0.8799	0.7526	0.4231

Western blot analysis

NE	control	surgery	surgery+nt-p65-TMD (5mg/kg)	surgery+nt-p65-TMD (10mg/kg)
Mean	1	1.232	0.9833	0.8481
Std. Error	0	0.0467	0.07534	0.05981
Iba-1	control	surgery	surgery+nt-p65-TMD (5mg/kg)	surgery+nt-p65-TMD (10mg/kg)
Mean	1	1.206	0.8503	0.7656
Std. Error	0	0.02665	0.03109	0.04918
CE11b	control	surgery	surgery+nt-p65-TMD (5mg/kg)	surgery+nt-p65-TMD (10mg/kg)
Mean	1	1.621	0.7414	0.8491
Std. Error	0	0.1656	0.03355	0.0687

Iba-1 expression

CA1	control	surgery	surgery+nt-p65-TMD (5mg/kg)	surgery+nt-p65-TMD (10mg/kg)
Mean	16.45	24.15	20.39	18.1
Std. Error	1.054	0.6188	1.309	1.757
CA3	control	surgery	surgery+nt-p65-TMD (5mg/kg)	surgery+nt-p65-TMD (10mg/kg)
Mean	13.48	24.31	15.57	15.5
Std. Error	0.8548	2.462	1.646	0.4631
DG	control	surgery	surgery+nt-p65-TMD (5mg/kg)	surgery+nt-p65-TMD (10mg/kg)
Mean	15.76	23.98	19.13	18.63
Std. Error	0.8272	1.606	0.4642	1.115

Table 8. Statistical parameter in Supplementary Figure 1.

VCAM-1 expression

CA1	control	surgery	surgery+nt-p65-TMD (5mg/kg)	surgery+nt-p65-TMD (10mg/kg)
Mean	15.81	26.01	16.94	15.9
Std. Error	0.0951	2.591	0.6943	0.6395

CA3	control	surgery	surgery+nt-p65-TMD (5mg/kg)	surgery+nt-p65-TMD (10mg/kg)
Mean	12.44	25.54	14.54	14.21
Std. Error	0.1989	3.785	0.1321	0.6782

DG	control	surgery	surgery+nt-p65-TMD (5mg/kg)	surgery+nt-p65-TMD (10mg/kg)
Mean	17.94	26.19	16.77	15
Std. Error	1.307	1.143	1.444	0.3221

ICAM-1 expression

CA1	control	surgery	surgery+nt-p65-TMD (5mg/kg)	surgery+nt-p65-TMD (10mg/kg)
Mean	30.21	44.65	32.35	35.89
Std. Error	0.4393	0.9887	0.7637	0.959

CA3	control	surgery	surgery+nt-p65-TMD (5mg/kg)	surgery+nt-p65-TMD (10mg/kg)
Mean	37.18	54.2	43.63	42.71
Std. Error	4.441	1.865	1.777	2.077

DG	control	surgery	surgery+nt-p65-TMD (5mg/kg)	surgery+nt-p65-TMD (10mg/kg)
Mean	35.46	50.65	43.5	42.56
Std. Error	2.125	0.8208	1.054	2.172

P-selectin expression

CA1	control	surgery	surgery+nt-p65-TMD (5mg/kg)	surgery+nt-p65-TMD (10mg/kg)
Mean	17.72	29.79	22.4	18.01
Std. Error	0.2876	3.457	1.367	1.251

CA3	control	surgery	surgery+nt-p65-TMD (5mg/kg)	surgery+nt-p65-TMD (10mg/kg)
Mean	13.16	24.04	22.33	15.99
Std. Error	0.3999	1.705	1.574	0.6261

DG	control	surgery	surgery+nt-p65-TMD (5mg/kg)	surgery+nt-p65-TMD (10mg/kg)
Mean	18.52	29.98	22.02	15.4
Std. Error	0.2074	1.775	1.59	0.6152

E-selectin expression

CA1	control	surgery	surgery+nt-p65-TMD (5mg/kg)	surgery+nt-p65-TMD (10mg/kg)
Mean	18.21	31.34	25.49	19.27
Std. Error	3.058	1.57	2.01	2.351

CA3	control	surgery	surgery+nt-p65-TMD (5mg/kg)	surgery+nt-p65-TMD (10mg/kg)
Mean	21.47	44.88	25.5	24.48
Std. Error	0.3607	5.289	2.383	2.591

DG	control	surgery	surgery+nt-p65-TMD (5mg/kg)	surgery+nt-p65-TMD (10mg/kg)
Mean	16.37	31.5	27.4	24.38
Std. Error	2.659	1.18	3.582	2.707

Supplementary Table 8. Statistical parameter in Supplementary Figure 1.
